# ZmnMAT1, a nuclear-encoded type I maturase, is required for the splicing of mitochondrial *Nad1* intron 1 and *Nad4* intron 2

**DOI:** 10.3389/fpls.2022.1033869

**Published:** 2022-11-23

**Authors:** Kaijian Fan, Qinghui Fu, Qianhan Wei, Sinian Jia, Anqi Zhao, Tengteng Wang, Jie Cao, Yan Liu, Zhenjing Ren, Yunjun Liu

**Affiliations:** ^1^ Institute of Crop Sciences, Chinese Academy of Agricultural Sciences, Beijing, China; ^2^ College of Agronomy and Biotechnology, China Agricultural University, Beijing, China; ^3^ College of Agronomy, Gansu Agricultural University, Lanzhou, Gansu, China; ^4^ State Key Laboratory of Dao-di Herbs, National Resource Center for Chinese Materia Medica, China Academy of Chinese Medical Sciences, Beijing, China; ^5^ College of Agronomy and Biotechnology, Yunnan Agricultural University, Kunming, Yunnan, China

**Keywords:** *ZmnMAT1*, group II intron splicing, type I maturase, mitochondrion, seed development, maize

## Abstract

Maturases can specifically bind to intron-containing pre-RNAs, folding them into catalytic structures that facilitate intron splicing *in vivo*. Plants possess four nuclear-encoded maturase-related factors (nMAT1-nMAT4) and some maturases have been shown to involve in the splicing of different mitochondrial group II introns; however, the specific biological functions of maturases in maize are largely uncharacterized. In this study, we identified a maize *ZmnMAT1* gene, which encodes a mitochondrion-localized type I maturase with an RT domain at N-terminus and an X domain at C-terminus. Loss-of-function mutation in *ZmnMAT1* significantly reduced the splicing efficiencies of *Nad1* intron 1 and *Nad4* intron 2, and showed arrested embryogenesis and endosperm development, which may be related to impaired mitochondrial ultrastructure and function due to the destruction of the assembly and activity of complex I. Direct physical interaction was undetectable between ZmnMAT1 and the proteins associated with the splicing of *Nad1* intron 1 and/or *Nad4* intron 2 by yeast two-hybrid assays, suggesting the complexity of group II intron splicing in plants.

## Introduction

Group II introns are large catalytic RNAs, which are prevalent in bacteria, organellar genomes of lower eukaryotes, and the mitochondrial genomes in plants (Lambowitz and Zimmerly, 2011; Brown et al., 2014). Canonical group II introns consist of a catalytic ribozyme and an intron-encoded maturase, which can self-splice *in vitro* in the absence of any cofactors under nonphysiologically conditions; or be assisted by *trans*-acting proteinaceous cofactors for the efficient splicing *in vivo* under physiological conditions (Ahlert et al., 2006). During evolution, the plant mitochondrial group II introns have undergone tremendous degeneration and divergence, resulting in loss of self-splicing function due to the lack of evolutionary related cognate intron-encoded maturase proteins (Shevtsov-Tal et al., 2021). As a result, only a single immobile maturase gene, *MatR* (locating in the *Nad1* intron 4), has been retained in the mitochondrial DNA (mtDNA) in angiosperms (Sultan et al., 2016). Intriguingly, in addition to *MatR*, plants also harbor another four nuclear-encoding maturase genes (named *nMAT1* to *nMAT4*) with mitochondrial-localized signals in their N-terminus (Brown et al., 2014). To facilitate the splicing and processing of group II introns in plant mitochondria, a variety of nuclear-encoded RNA-binding factors belonging to different protein families, including pentatricopeptide repeat (PPR) proteins, chloroplast RNA splicing and ribosome maturation (CRM) proteins, mitochondrial transcription termination factors (mTERF), plant organellar RNA recognition (PORR) proteins, RNA helicase, RAD52-like proteins, and regulator of chromosome condensation (RCC) domain proteins are recruited (Brown et al., 2014; Fan et al., 2021).

Typical nuclear-encoded maturases are characterized by three conserved functional domains: a N-terminal reverse transcriptase (RT) domain, a RNA binding and splicing (X) domain, and a catalytic C-terminal DNA binding (D) and endonuclease domain (En) (Cohen et al., 2014). According to the topological structures and evolutionary relationships, the four nMATs are further divided into type I (including nMAT1 and nMAT2 with RT and X domains) and type II (containing nMAT3 and nMAT4 with RT, X and D-En domains) maturases (Mohr and Lambowitz, 2003). However, the alterations in RT and D-En motifs of the four nMATs suggests an expected lack of mobility-associated functions and endonuclease activities in angiosperms (Mohr and Lambowitz, 2003). MatR is associated with the splicing of various group II introns including *Nad1* intron 1, 3 and 4 (the host intron of *MatR*), *Nad4* intron 1, *Nad5* intron 4, *Nad7* intron 2, *Rpl2* intron 1, and *Rps3* intron 3 in Brassicaceae mitochondria (Sultan et al., 2016). In Arabidopsis, nuclear-encoded AtnMAT1 functions in the splicing of *Nad1* intron 1, *Nad2* intron 1 and *Nad4* intron 2; homozygous *atnmat1* plants showed impaired assembly and activity of mitochondrial complex I, leading to retarded growth and development (Keren et al., 2012). The mutation in *AtnMAT2* affected the splicing efficiency of *Nad1* intron 2, *Nad7* intron 2 and the single intron within *Cox2*, resulting in defective vegetative growth and floral meristem development (Keren et al., 2009). For nMAT3, Arabidopsis AtnMAT3 is required for the splicing of *Nad1* intron 1, 3 and 4, as well as *Nad2* intron 1 and 2 (Shevtsov-Tal et al., 2021). Similarity, maize ZmnMAT3 is particularly required for the splicing of *Nad1* intron 1, 3 and 4, and also affects the splicing efficiency of *Nad2* intron 2, *Nad5* intron 1 and 2, and *Nad7* intron 1 (Chen et al., 2021). Loss-of-function of AtnMAT3 or ZmnMAT3 led to defective complex I activity and retarded embryogenesis (Chen et al., 2021; Shevtsov-Tal et al., 2021). In addition, AtnMAT4 is essential for RNA processing and maturation of *Nad1* introns 1, 3 and 4, as well as seed germination, seedling establishment and development in Arabidopsis (Cohen et al., 2014). However, the function of most maturases (including ZmMatR, ZmnMAT1, ZmnMAT2 and ZmnMAT4) are still unknown in maize.

In this study, we characterized a mitochondrion-localized type I nuclear-encoded maturase, ZmnMAT1, which was found to be required for the *trans*-splicing of *Nad1* intron 1 and *cis*-splicing of *Nad4* intron 2. Mutation in ZmnMAT1 showed arrested embryogenesis and endosperm development, which may be associated with the damage of assembly and activity of complex I. We further confirmed that no direct physical interaction was detected between ZmnMAT1 and the proteins related to the splicing of *Nad1* intron 1 and/or *Nad4* intron 2 by yeast two-hybrid assays, suggesting a complex mechanism of group II intron splicing in maize.

## Results

### Loss of function of *ZmnMAT1* produces empty pericarp kernels with severely arrested development

**Figure 1:**
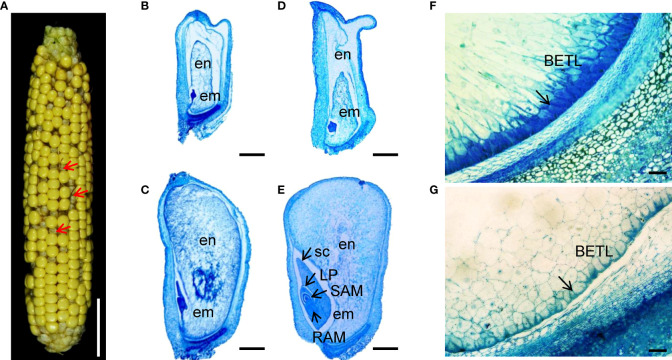
Embryo and endosperm development is arrested in the *zmnmat1* mutant. **(A)** *zmnmat1* mutant ears at 15 DAP. Red arrows indicate the empty pericarp kernels. Scale bar =4 cm. **(B-E)** Paraffin sections of 10 DAP **(B, C)** and 15 DAP **(D, E)** WT **(C, E)** and mutant **(B, D)** kernels from F_2_ ears crossed by *zmnmat1* and B73. Arrows indicate the embryo. em, embryo; en, endosperm; LP, leaf primordia; RAM, root apical meristem; SAM, shoot apical meristem; sc, scutellum. Scale bar =1 mm. **(F, G)** The BETL cells of 15 DAP WT **(F)** and *zmnmat1* mutant kernel **(G)**. Arrows indicate the BETL cells. Scale bar=50 μm.

Maize *zmnmat1* is an empty pericarp (emp) mutant derived from the UniformMu stock under accession number UFMu-05745 (McCarty et al., 2005). The *zmnmat1*/*+* plants were crossed into the B73 genetic background to produce F_1_ population. F_2_ ears from the self-pollinated F_1_ progenies with *zmnmat1*/+ heterozygotes displayed a ratio of 3:1 (+/+ and *zmnmat1*/*+*: *zmnmat1*/*zmnmat1*, 1927:659, χ^2^=0.30), indicating that *zmnmat1* is a monogenic and recessive mutant (**Figure 1A**; **Supplementary Figure S1A**). The homozygous *zmnmat1* kernels harbor smaller, white and vitreous endosperms and could be easily distinguished from their wild-type (Clifton et al.) siblings from 10 days after pollination (DAP). At 15 DAP, the phenotype of mutant kernels became more striking with wrinkled pericarp (**Figure 1A**). At maturity, homozygous *zmnmat1* kernels were further collapsed, resulting in an unviable *emp* phenotype (**Supplementary Figure S1A**).

To investigate the impact of *ZmnMAT1* mutation on embryogenesis and endosperm development, *zmnmat1* and WT kernels from the same segregating ear at different developmental stage were sectioned and analyzed. At 10 DAP, the embryos of WT kernels had already reached the coleoptilar stage characterized by obvious scutellum (**Figure 1C**), while the *zmnmat1* embryos were still blocked at the transition stage with smaller size (**Figure 1B**). At 15 DAP, the WT embryos had developed to the late embryogenesis stage with well-differentiated scutellum (sc), leaf primordia (LP), shoot apical meristem (SAM), and root apical meristem (RAM) (**Figure 1E**), whereas the *zmnmat1* embryos remained at the transition stage with visible undifferentiated embryos (**Figure 1D**). In addition, the endosperm development was also inhibited in *zmnmat1* mutant during the developmental profile, with much smaller size, incompact starch-packed and a large interspace between seed coat and endosperm (**Figures 1B-E**). These results indicate that the mutation of *ZmnMAT1* severely affected embryo and endosperm development.

In addition, basal endosperm transfer layer (BETL) cells were also examined at 15 DAP. The BETL cells of wild-type kernels showed obvious cell wall ingrowths (**Figure 1F**), while the BETL cells in *zmnmat1* kernels were almost invisible (**Figure 1G**). These results suggest that the defect of *zmnmat1* kernels may result from abnormal BETL cells, which function in nutrient transport from maternal tissue into kernel endosperm cells.

### Identification of the *ZmnMAT1* gene

**Figure 2:**
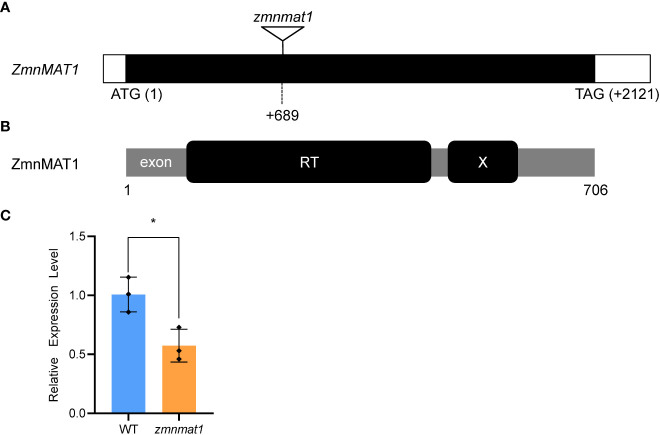
Cloning and identification of the *ZmnMAT1* gene. **(A)** Structure of the *ZmnMAT1* gene and positions of *Mu* insertion (triangle) in *zmnmat1* mutant. **(B)** The ZmnMAT1 protein contains a RT motif (a reverse transcriptase domain) and an X motif (an RNA binding and splicing domain). **(C)** qRT-PCR analysis of *ZmnMAT1* expression in 10 DAP kernels of *zmnmat1* mutant and WT siblings. Expression level was normalized against *ZmActin*. Values are shown as the average ± S.E. (* *p*< 0.05, Student’s *t*-test).

To clone the causal gene, the *Mu*-flanking PCR was conducted and sequenced subsequently to identify the insertion site of *Mu* element in *zmnmat1* mutant (Liu et al., 2016). Sequence analysis indicated that a *Mu* transposon located at +689 bp downstream from the translation start codon of a putative maturase-related gene *GRMZM2G023983*, so the annotated gene was named as *ZmnMAT1* (**Figure 2A**). Quantitative real-time PCR (qRT-PCR) analysis showed that the transcriptional level of *GRMZM2G023983* in *zmnmat1* kernels at 10 DAP was down-regulated significantly (**Figure 2C**). Linkage analysis based on the genotypes of 134 F_1_ plants and the phenotypes of their F_2_ ears was performed to confirm whether the mutation in *GRMZM2G023983* caused the *emp* phenotype. As *zmnmat1* homozygotes were embryo-lethal, only heterozygotes (*zmnmat1*/+; segregating) or the WT (+/+; non-segregating) were available for this analysis. As a result, only the F_1_ plants with *Mu* element insertion produced F_2_ ears with mutant kernels (**Supplementary Figure S2**) and the *Mu* element insertion site co-segregated with the *emp* phenotype (*zmnmat1*/+:+/+, 69:65, corresponding to 1:1 ratio, χ^2^=0.07), suggesting that *GRMZM2G023983* is the causative gene for *ZmnMAT1*. To further confirm that *GRMZM2G023983* was *ZmnMAT1*, targeted mutagenesis of *GRMZM2G023983* was performed using the CRISPR/Cas9 system. Two independent homozygous edited lines for the *GRMZM2G023983* gene named *zmnmat1-cas9-9* (edited with a 205 bp deletion between the two target sites) and *zmnmat1-cas9-18* (edited with a 229 bp deletion between the two target sites) were detected, unfortunately, we failed to obtain the final plants due to severe developmental stunting of these homozygous edited plants (**Supplementary Figure S3**).

### *ZmnMAT1* encodes a mitochondrion-localized type I maturase

**Figure 3:**
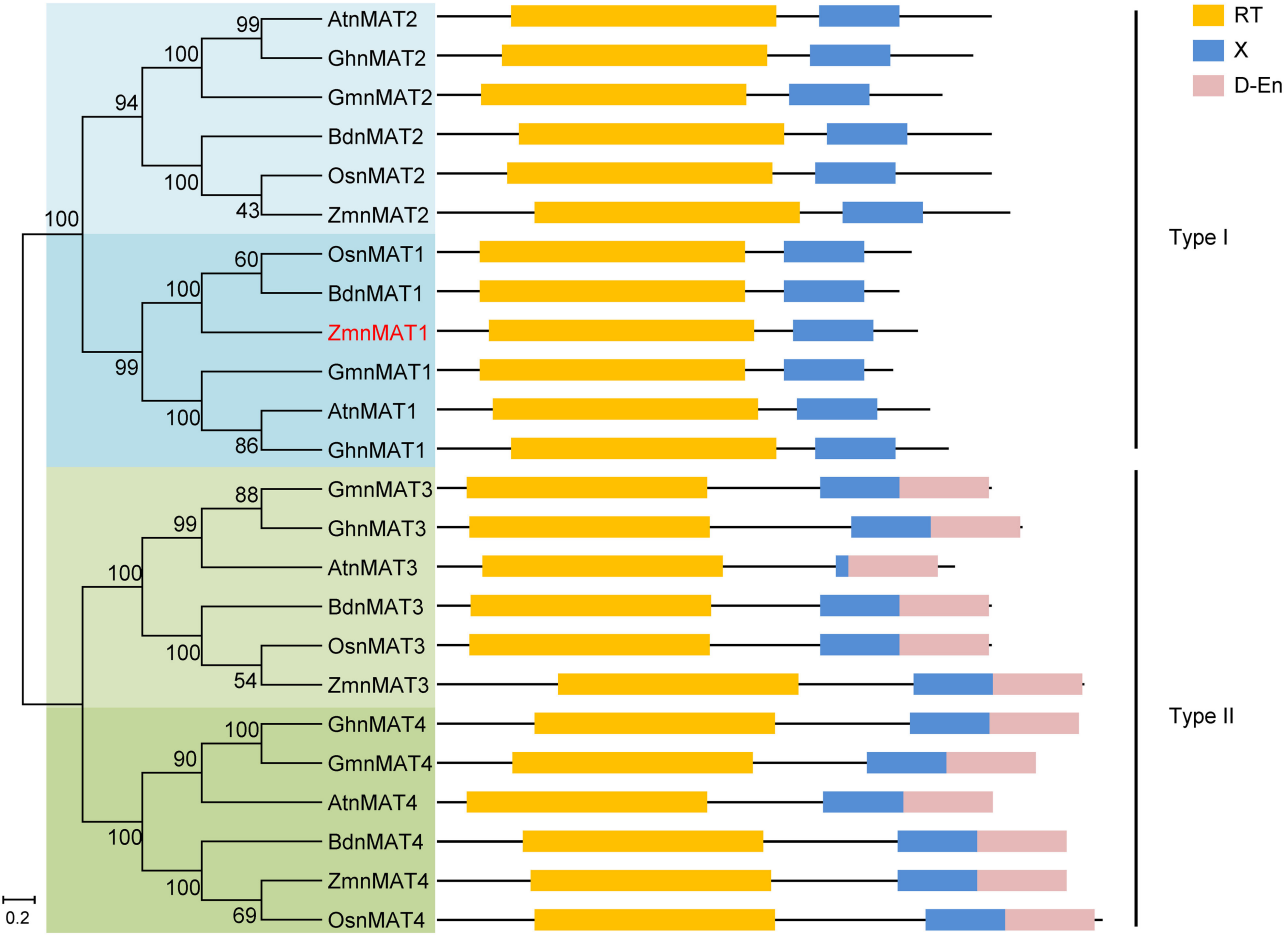
Phylogenetic relationships and protein motifs of ZmnMAT1 and its homologs. The ZmnMAT1 protein and its homologs from NCBI were aligned by ClustalW, and the phylogenetic tree was constructed using MEGA 5.0 software. Distances were estimated with a neighbor-joining algorithm, and bootstrap support is indicated to the left of branches. Scale bar indicates the average number of amino acid substitutions per site. RT, X and D-En motifs represent the reverse transcriptase domain, an RNA binding and splicing domain, and DNA-binding endonucleases domain, respectively.

According to the B73 reference genome (Schnable et al., 2009), the full-length cDNA sequence of *ZmnMAT1* was amplified from inbred line B73. Sequence alignment revealed that *ZmnMAT1* contains a 2121 bp long open reading frame (ORF) with no intron and is predicted to a maturase-related protein of 706 amino acids (**Figures 2A, B**). Previous studies has showed that the classic type II maturases are characterized by three conserved functional domains named RT, X, and D-En, while the canonical type I maturases only contain RT and X domains without D-En domain (Shevtsov-Tal et al., 2021). A phylogenetic tree including twenty-four ZmnMAT1 protein homologs from *Zea mays* (ZmnMAT1 to ZmnMAT4), *Arabidopsis thaliana* (AtnMAT1 to AtnMAT4), *Oryza sativa* (OsnMAT1 to OsnMAT4), *Glycine max* (GmnMAT1 to GmnMAT4), *Brachypodium distachyon* (BdnMAT1 to BdnMAT4), and *Gossypium hirsutum* (GhnMAT1 to GhnMAT4) was constructed. The result revealed two major clades: nMAT1 and nMAT2 belong to type I maturases, and nMAT3 and nMAT4 belong to type II maturases (**Figure 3**); which is in accordance with the previously prediction based on topology and evolutionary origins (Cohen et al., 2014). ZmnMAT1 belongs to the type I maturases with RT and X domains and is more closely related to monocotyledonous OsnMAT1 and BdnMAT1 (**Figure 3**). Further, a detailed sequence alignment among ZmnMAT1 and its orthologs (AtnMAT1, OsnMAT1, GmnMAT1, BdnMAT1 and GhnMAT1) indicated that ZmnMAT1 share a high degree of conserved RT and X domains (**Supplementary Figure S4**).

**Figure 4:**
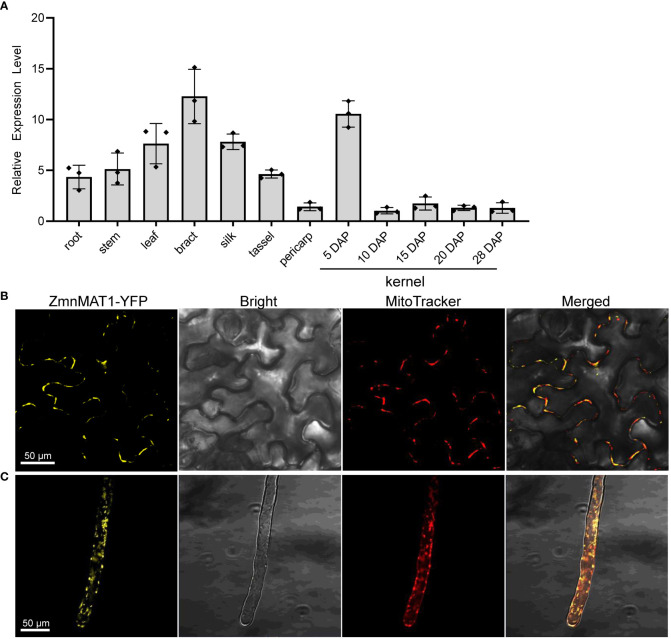
The expression profile of *ZmnMAT1* and subcellular localization of ZmnMAT1 protein. **(A)** Expression profiles of *ZmnMAT1* in various tissues and during maize kernel development. *ZmActin* was used as an internal control. Data was shown as the average ± S.E. of three biological replicates. **(B)** Subcellular localization of 35S:ZmnMAT1-YFP fusion protein in tobacco leaf epidermis cells. MitoTracker Red was used to label mitochondria. Scale bar =50 μm. **(C)** Subcellular localization of 35S:ZmnMAT1-YFP fusion protein in *Arabidopsis thaliana* root hairs. MitoTracker Red was used to label mitochondria. Scale bar =50 μm. Fluorescence of YFP and MitoTracker were detected with excitation at 514 and 579 nm, respectively. And the master gain of YFP and MitoTracker were 731 and 500, respectively.

The expression profile of *ZmnMAT1* was examined by qRT-PCR with multiple tissues from inbred line B73. *ZmnMAT1* was detected to be constitutively expressed in all tested vegetative and reproductive tissues, with relatively higher transcriptional level in bract and kernels at 5 DAP, and lower expression level in pericarp and kernels at late developing stage (**Figure 4A**). These results suggest that *ZmnMAT1* may affect several aspects of plant growth and development.

To investigate the cellular localization of ZmnMAT1 protein, the full-length *ZmnMAT1* coding sequence without stop codon was fused to the N-terminus of YFP, generating a p35S::ZmnMAT1-YFP vector, which was then transiently expressed in leaf epidermal cells of *Nicotiana benthamiana*, or transformed stably into Arabidopsis. Both yellow fluorescence signals of ZmnMAT1-YFP from leaf epidermal cells and root hairs in punctuated spots were overlapped with the MitoTracker Red (a mitochondrion-labelled dye), suggesting that ZmnMAT1 is targeted to the mitochondria (**Figures 4B, C**).

### ZmnMAT1 is required for the *trans*-splicing of *Nad1* intron 1 and *cis*-splicing of *Nad4* intron 2

**Figure 5:**
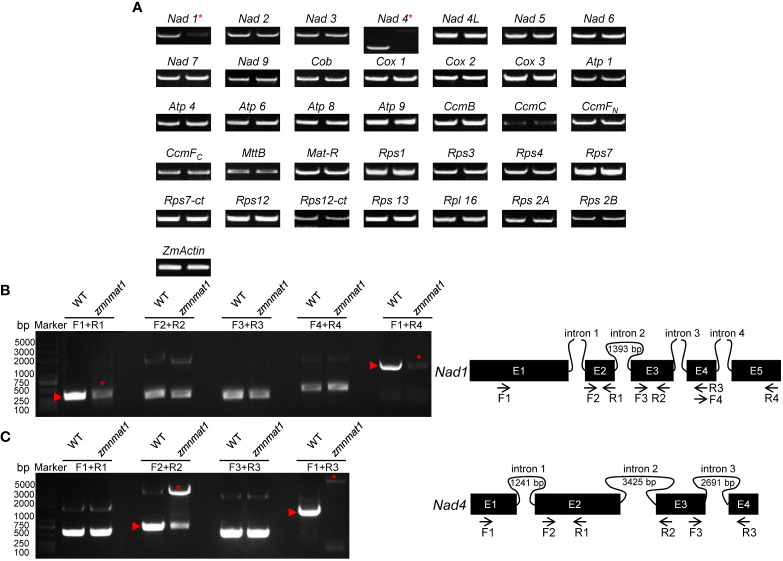
The loss of *Nad1* and *Nad4* mature transcripts and splicing deficiency of *Nad1* intron 1 and *Nad4* intron 2 in *zmnmat1* mutant. **(A)** RT-PCR analysis of 35 mitochondria-encoded transcripts in 10 DAP WT (left) and *zmnmat1* mutant (right) kernels. *ZmActin* was used as internal control. **(B)** RT-PCR analysis of *Nad1* intron-splicing efficiency in WT and *zmnmat1* mutants (left) and schematic structure of *Nad1* gene (right). The expected amplification products using different primer pairs are indicated (right). Red asterisks indicate the abnormal unspliced fragments and red triangles indicate the normal spliced fragments (left). **(C)** RT-PCR analysis of *Nad4* intron-splicing efficiency in WT and *zmnmat1* mutants (left) and schematic structure of *Nad4* gene (right). The expected amplification products using different primer pairs are indicated (right). Red asterisks indicate the abnormal unspliced fragments and red triangles indicate the normal spliced fragments (left).

It has been shown that nMAT proteins function in the splicing of mitochondrial group II introns in angiosperms (Brown et al., 2014). Thus, we firstly compared the expression levels of 35 mitochondrion-encoding genes between WT and the *zmnmat1* kernels at 10 DAP by RT-PCR. The results revealed that the mature transcripts of most genes were comparable between WT and the *zmnmat1* kernels, except for *Nad1* and *Nad4*, which were dramatically decreased or completely undetectable in *zmnmat1* kernels, respectively (**Figure 5A**), indicating a defective RNA processing in *Nad1* and *Nad4* precursor transcripts in *zmnmat1* mutant.

**Figure 6:**
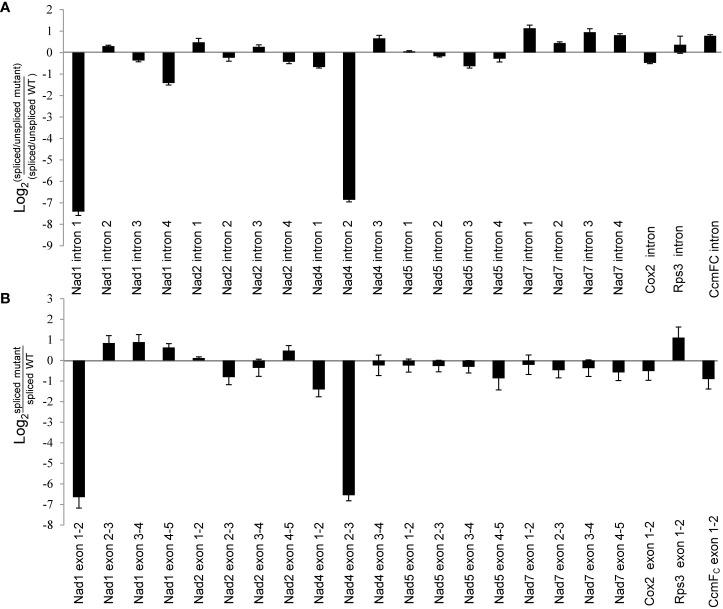
Splicing deficiency of *Nad1* intron 1 and *Nad4* intron 2 in 10 DAP *zmnmat1* kernels. **(A)** qRT-PCR analysis of intron splicing deficiency of mitochondrial genes. Primers spanned adjacent exons and introns were used to measure the differences in splicing efficiency. Data was shown as the average ± S.E. of three biological replicates. **(B)** qRT-PCR analysis of mature mitochondrial transcripts. Primers spanning adjacent exons were used for measuring differences in each spliced fragment. *ZmActin* was used as an internal control. Data was shown as the average ± S.E. of three biological replicates.

Maize mitochondrial *Nad1* contains three *trans*-splicing introns (intron 1, 3 and 4) and one *cis*-splicing intron 2, and *Nad4* contains three *cis*-splicing introns (**Figures 5B, C**). We subsequently analyzed the intron splicing events of *Nad1* and *Nad4*, as well as other genes (*Nad2*, *Nad5*, *Nad7*, *Cox2*, *Rps3*, and *CcmF_C_*) in WT and *zmnmat1* kernels by RT-PCR and qRT-PCR, to determine whether the down-regulation of mature *Nad1* and *Nad4* transcripts is a result of intron-splicing deficiency. The results showed that except for the decreased splicing efficiency of *Nad1* intron 1 and *Nad4* intron 2 in *zmnmat1* kernels (**Figures 5B, C**), no obvious differences were detected in the rest of 20 group II introns (**Supplementary Figure S5**), suggesting the requirement of *ZmnMAT1* in the *trans*-splicing of *Nad1* intron 1 and *cis*-splicing of *Nad4* intron 2. The partially reduced splicing of *Nad4* intron 2 resulted in the remaining of 3425 bp intron 2 in the *Nad4* mature transcripts of *zmnmat1* kernels (**Figure 5A**, **C**). Consistently, the intron splicing efficiency of *Nad1* intron 1 and *Nad4* intron2 were also affected in *zmnmat1-cas9-9* and *zmnmat1-cas9-18* mutants (**Supplementary Figure S6**). However, it cannot be confirmed that the empty pericarp phenotype is the result of such defective splicing, because these homozygous edited plants are severely developmental stunted and thus lethal. Furthermore, the PCR analysis with primer pairs across adjacent exons and introns showed dramatic reduction of splicing efficiency of both *Nad1* intron 1 and *Nad4* intron 2 in *zmnmat1* kernels, compared with that in the WT kernels (**Figure 6A**). The quantitative differences of spliced exons with primer pairs across adjacent exons were further examined, and the results showed that both the *Nad1* spliced exon 1-2 fragment and *Nad4* spliced exon 2-3 fragment were reduced about 128 times in *zmnmat1* mutant (**Figure 6B**). Compared with the WT, the splicing efficiency and spliced exons of *Nad2*, *Nad5*, *Nad7*, *Cox2*, *Rps3*, and *CcmF_C_* were unaffected in *zmnmat1* mutant (**Figures 6A, B**). These results demonstrate that ZmnMAT1 is a key nuclear-encoded splicing factor, which is involved in the splicing of *Nad1* intron 1 and *Nad4* intron 2 in maize mitochondria.

### The assembly and activity of mitochondrial complex I were severely impaired in *zmnmat1* mutant

**Figure 7:**
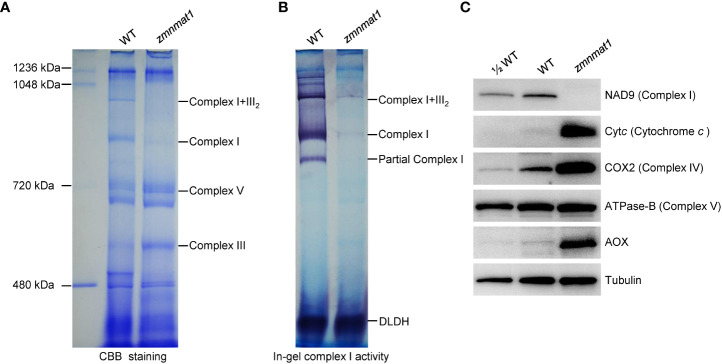
Impacted mitochondrial complexes in *zmnmat1* kernels at 10 DAP. **(A)** BN-PAGE analysis of mitochondrial complexes. The positions of complex I+III_2_, complex I, complex III and complex V were indicated. About 100 μg of mitochondrial protein was loaded in each lane. **(B)** In-gel NADH dehydrogenase activity test of complex (I) The positions of complex I+III_2_ and complex I was indicated. About 100 μg of mitochondrial protein was loaded in each lane. **(C)** Western blot analysis with antibodies against NAD9 (subunit of complex I), Cyt*c* (Cytochrome *c*), COX2 (subunit of complex IV), ATPase-B (subunit of complex V) and AOX2 (stands for alternative oxidase) in mitochondrial protein from WT and *zmnmat1* immature seeds at 10 DAP. Tubulin was used as a loading control. About 25 μg of mitochondrial protein was loaded in WT and *zmnmat1* lanes, and about 12.5 μg of mitochondrial protein was loaded in ½WT lane.

The mitochondrial *Nad1* and *Nad4* genes encode subunits NAD1 and NAD4, respectively, which are the components of complex I, an entry complex of the oxidative phosphorylation electron transfer chain (Clifton et al., 2004). Hence, defects in the posttranscriptional processing of *Nad1* and *Nad4* are likely to affect the assembly, activity and stability of complex I. Blue native polyacrylamide gel electrophoresis (BN-PAGE) and in-gel NADH dehydrogenase activity assay were performed to investigate the potential impact on the assembly and activity of complex I. Compared with the WT, the abundance of complex I and super complex I+III_2_ were strongly decreased (**Figure 7A**), indicating that the assembly of complex I was indeed affected in *zmnmat1* mutant. Interestingly, the abundance of complex III was markedly increased, which could be explained by a feedback regulation mode as reported previously (Ren et al., 2019). In addition, the NADH dehydrogenase activities of complex I and super complex I+III_2_ were significantly reduced in *zmnmat1* mutant (**Figure 7B**).

The stability of several respiratory chain complex-related proteins, including NAD9 (a subunit of complex I), Cyt*c* (cytochrome *c*), COX2 (a subunit of complex IV), and ATPase-B (a subunit of complex V) were further investigated by western blotting. The results showed that the abundance of NAD9 protein was greatly reduced in *zmnmat1* mutant (**Figure 7C**), suggesting that the reduced splicing efficiency in *zmnmat1* mutant caused a defect in the steady-state levels of complex-related protein. In contrast, the levels of Cyt*c* and COX2 proteins were significantly increased in *zmnmat1* mutant, and no remarkable change was detected in the level of ATPase-B protein between WT and *zmnmat1* mutant (**Figure 7C**). Taken together, these results suggest that the defective splicing in *Nad1* and *Nad4* are the direct cause of the decreased assembly, activity and stability of mitochondrial complex I in *zmnmat1* mutant.

### The alternative oxidase pathway was induced with altered mitochondrial ultrastructure in *zmnmat1* mutant

**Figure 8:**
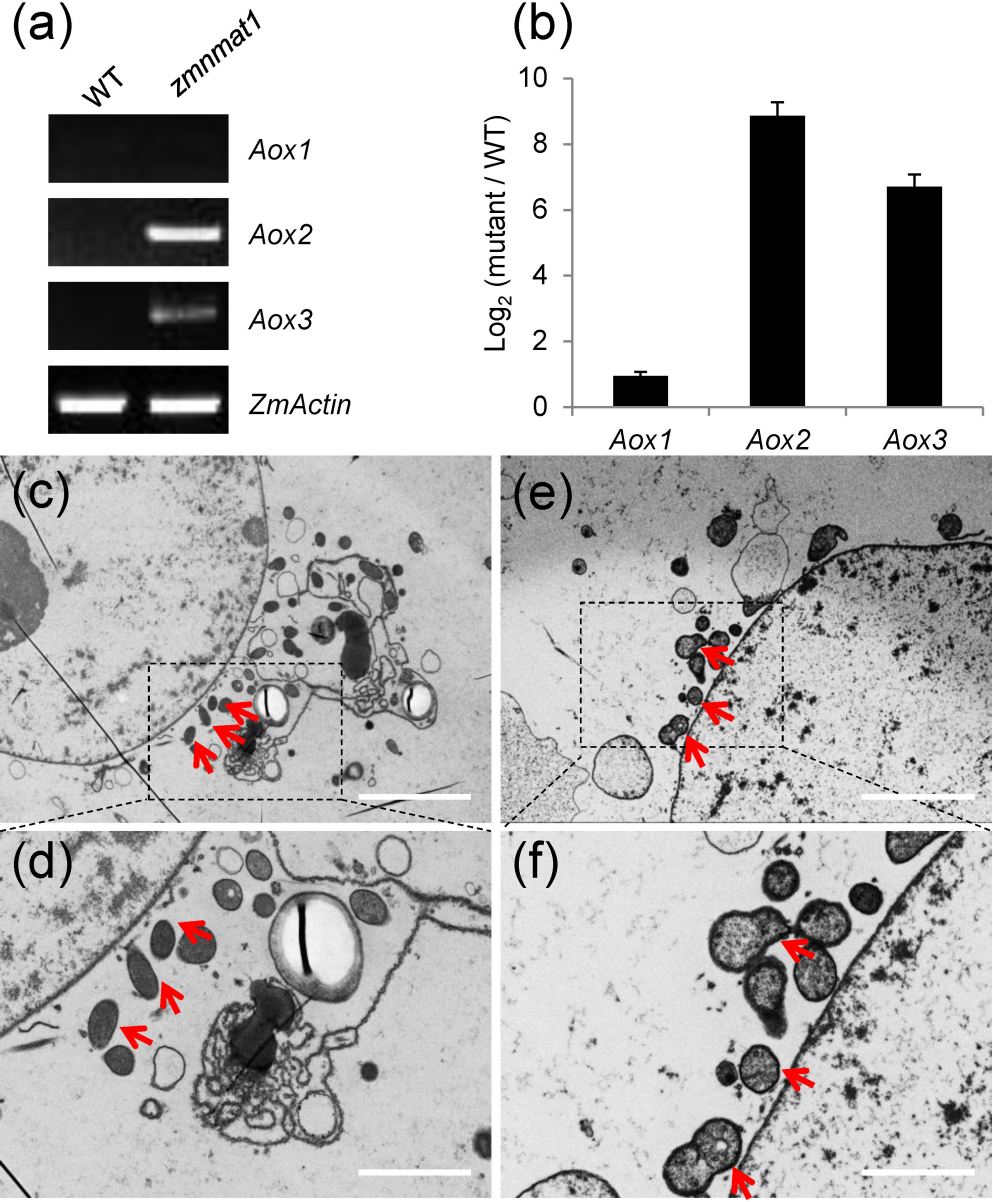
Expression of *Aox1*, *Aox2*, and *Aox3* in the *zmnmat1* mutant. **(A)** RT-PCR analysis of *Aox1*, *Aox2*, and *Aox3* expression in the 10 DAP WT and *zmnmat1* kernels. The expression level was normalized against *ZmActin*. **(B)** qRT-PCR analysis of *Aox1*, *Aox2*, and *Aox3* expression using *ZmActin* as an internal control. Data is the average ± S.E. of three biological replicates. **(C-F)** Transmission electron microscopy image of the WT **(C, D)** and *zmnmat1* mutant **(E, F)** in 15 DAP endosperm cells**. (C, E)** Low magnification. Scale bar=5 μm. **(D, F)** High magnification. Scale bar=2 μm. Red arrows indicate the mitochondria.

Plant mitochondrial respiratory chain contains two pathways: the main cytochrome respiratory pathway and a branched alternative oxidase pathway (Vanlerberghe, 2013). Usually, the blockage of cytochrome respiratory pathway will lead to activated alternative compensatory pathway (Vanlerberghe, 2013; Cai et al., 2017; Sun et al., 2018). In *zmnmat1* mutant, the defective assembly and activity of complex I inhibited the normal function of cytochrome respiratory pathway. Thus, the transcriptional and protein levels of alternative oxidase (*Aox*) genes were examined to check whether the alternative pathway was induced. The results showed ~ 2-fold, 512-fold and 128-fold increase in the expression levels of *Aox1*, *Aox*2 and *Aox*3 genes in *zmnmat1* mutant, compared with the WT, respectively (**Figures 8A, B**). Three maize AOX proteins have high sequence similarity, and they are undistinguishable by the polyclonal antibody. Consistently, AOX protein level was also dramatically accumulated in the *zmnmat1* mutant by western blotting (**Figure 7C**). In summary, these results indicate that the mutation in ZmnMAT1 interrupts the cytochrome respiratory pathway and increases gene expression of the alternative pathway in maize mitochondria.

To investigate the impact of *zmnmat1* on mitochondrial morphology, ultra-thin sections of 15 DAP endosperm from *zmnmat1* and the WT kernels were observed by transmission electron microscopy (TEM). The WT endosperm exhibited normal mitochondria with dense texture (**Figures 8C, D**), whereas the mitochondria of *zmnmat1* showed a poorly developed membrane system with large internal spaces (**Figures 8E, F**), indicating that ZmnMAT1 is required for the proper structure and function of mitochondria during seed development.

### ZmnMAT1 might not directly interact with the proteins involved in the splicing of *Nad1* intron 1 and/or *Nad4* intron 2

Several research provided pieces of evidence that nuclear-encoded splicing factors may cooperate in regulating the splicing of one or more specific introns by forming possible spliceosomal complexes (de Longevialle et al., 2010; Fan et al., 2021). It is reported that maturase AtnMAT2 has been found in a large ribonucleoprotein complex in Arabidopsis mitochondria, which also contains RNA helicase PMH2 (Zmudjak et al., 2017). In maize, five proteins have been reported to be involved in the *trans*-splicing of *Nad1* intron 1, including DEK2 (Qi et al., 2017), EMP11 (Ren et al., 2017), PPR-SMR1 (Chen et al., 2019), DEK55 (Ren et al., 2020), and ZmnMAT3 (Chen et al., 2021); and only PPR-SMR1 was reported to be participated in the *cis*-splicing of *Nad4* intron 2. In Arabidopsis, except for PPR protein OTP43 (de Longevialle et al., 2007), four nuclear-encoded factors from three diverse families named AtnMAT1 (Keren et al., 2012) and AtnMAT4 (Cohen et al., 2014) of maturase family, ABO6 (He et al., 2012) of helicase family, and CFM9 (Lee et al., 2019) of CRM domain-containing family, have been reported to be required for the splicing of *Nad1* intron 1; in addition, AtnMAT1, ABO6 and PMH2 (Köhler et al., 2010) have been identified to take part in the splicing of *Nad4* intron 2. We speculated whether the orthologs of AtnMAT4, ABO6, CFM9, and PMH2 participate in the splicing of the *Nad1* intron 1 and/or *Nad4* intron 2 in maize.

**Figure 9:**
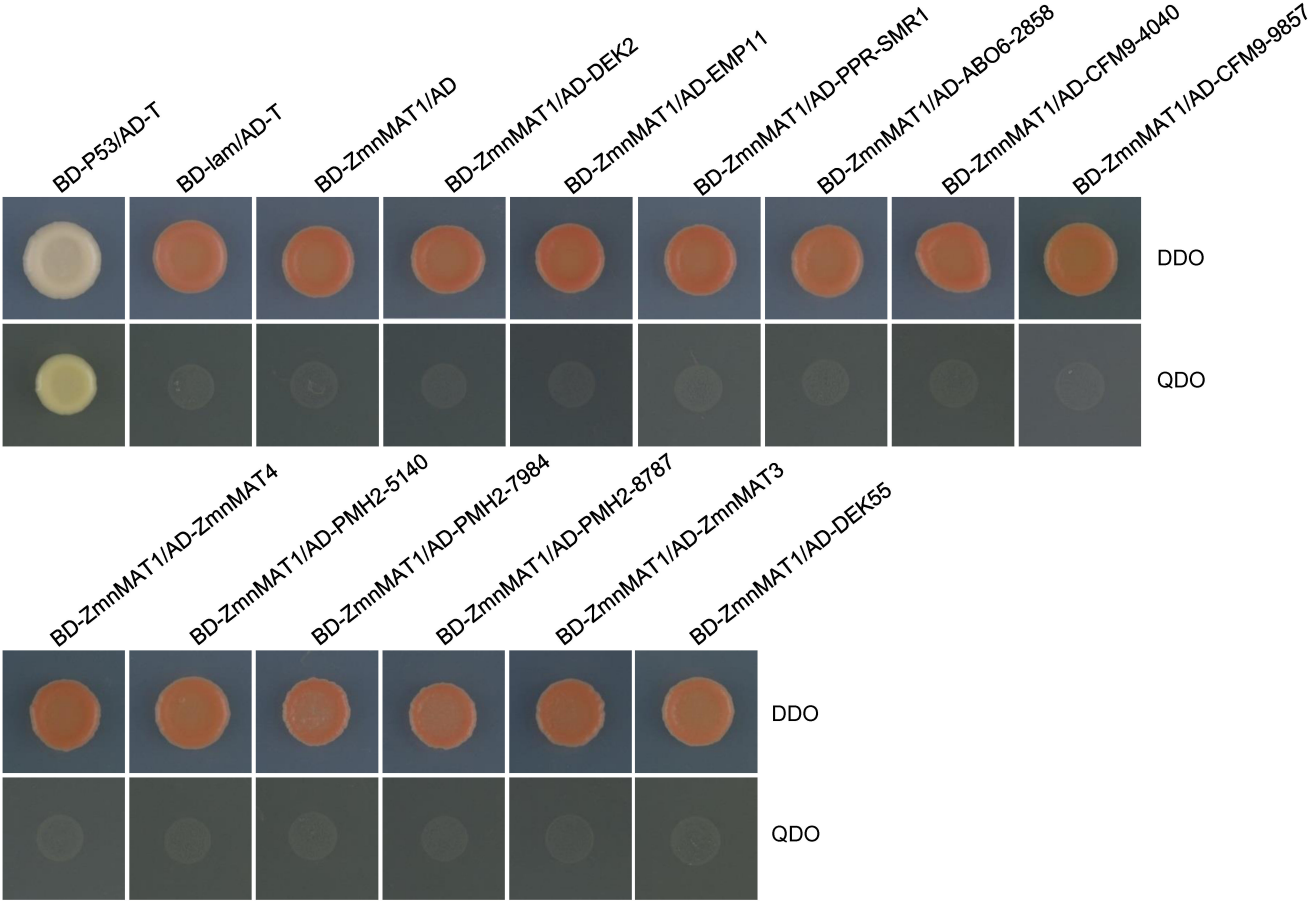
Y2H analysis of the interaction between ZmnMAT1 with factors involved in the splicing of *Nad1* intron 1 and/or *Nad4* intron 2. The Y2H strains harboring the indicated bait and prey constructs were spotted on SD-Trp-Leu (DDO) and SD-Trp-Leu-His-Ade (QDO).The interaction between T-antigen and Human P53 was used as a positive control. The interaction between T-antigen/Lam and BD-ZmnMAT1/AD were used as negative controls. AD, activation domain; BD, binding domain.

BLAST analysis indicated that the maize orthologs of AtnMAT4, ABO6, CFM9, and PMH2 are GRMZM2G375999 (named ZmnMAT4); GRMZM5G802858 (named ABO6-2858); GRMZM2G054040 (named CFM9-4040) and GRMZM2G039857 (named CFM9-9857); GRMZM2G565140 (named PMH2-5140), GRMZM2G080512 (named PMH2-0512), and GRMZM2G107984 (named PMH2-7984), respectively. Yeast two-hybrid assays were performed to test whether ZmnMAT1 could interact with these 12 putative splicing factors (including DEK2, EMP11, PPR-SMR1, DEK55, ZmnMAT3, ZmnMAT4, ABO6-2858, CFM9-4040, CFM9-9857, PMH2-5140, PMH2-0512, and PMH2-7984). Unfortunately, no direct interaction was detected between ZmnMAT1 and any of these proteins by Y2H assays (**Figure 9**).

## Discussion

### Differences in the splicing of specific introns of nMAT1 between maize and Arabidopsis

Arabidopsis AtnMAT1, a nuclear-encoded type I maturase, has been reported to be required for the splicing of three mitochondrial group II introns, including *Nad1* intron 1, *Nad2* intron 1 and *Nad4* intron 2 (Keren et al., 2012). In this study, we found that ZmnMAT1, an ortholog of AtnMAT1, is essential for the *trans*-splicing of *Nad1* intron 1 and *cis*-splicing of *Nad4* intron 2, but not for *Nad2* intron 1. Similarly, Arabidopsis AtnMAT3 is required for the splicing of *Nad1* intron 1, 3 and 4, as well as *Nad2* intron 1 and 2 (Shevtsov-Tal et al., 2021). By contrast, in maize, ZmnMAT3 is particularly required for the splicing of *Nad1* intron 1, 3 and 4, which also affects the splicing efficiency of *Nad2* intron 2, *Nad5* intron 1 and 2, and *Nad7* intron 1 (Chen et al., 2021). Analysis of the evolutionary relationship of type I maturase (nMAT1) among different species revealed a high conservativeness, especially in monocotyledons (**Figure 3**). Moreover, alignment of ZmnMAT1, AtnMAT1, OsnMAT1, GmnMAT1, BdnMAT1, and GhnMAT1 showed a high amino acid sequence similarity between ZmnMAT1 and monocotyledonous OsnMAT1 and BdnMAT1, especially in RT and X motifs (**Supplementary Figure S4**), suggesting a possible more conserved function of ZmnMAT1 in monocots. The distinct disparity of maturases in the splicing of specific introns between monocots and discots, may reflect the evolutionary divergence and complexity of intron splicing in plant mitochondria.

The nuclear genomes of angiosperms harbor four mitochondrial-localized maturase genes named *nMAT1* to *nMAT4* (Brown et al., 2014). In maize, four nuclear-encoding maturase genes (*ZmnMAT1*, *ZmnMAT2*, *ZmnMAT3* and *ZmnMAT4*) were also found according to the similarity of corresponding amino acid sequences. Among them, ZmnMAT1 is required for the splicing of *Nad1* intron 1 and *Nad4* intron 2; ZmnMAT3 is essential for the splicing of *Nad1* intron 1, 3 and 4, *Nad2* intron 2, *Nad5* intron 1 and 2, and *Nad7* intron 1 (Chen et al., 2021). ZmnMAT1 and ZmnMAT3 act on the same RNA target *Nad1* intron 1, however, mutation in either ZmnMAT1 or ZmnMAT3 leads to defective maize seed development with empty pericarp phenotype, suggesting that functions of ZmnMAT1 and ZmnMAT3 in the splicing of *Nad1* intron 1 are not redundant. Bioinformatics analysis failed to reveal the existence of common motifs or conservative structural features that could explain the specificities of different nMATs to their genetically recognized pre-RNAs (Keren et al., 2009; Cohen et al., 2014; Schmitz-Linneweber et al., 2015). In this study, mutation of ZmnMAT1 affected the splicing of mitochondrial *Nad1* intron 1 and *Nad4* intron 2; however, the direct links between ZmnMAT1 and its interactive RNA targets *in vivo* are still unknown. To solve this problem, RNA immunoprecipitation and high-throughput sequencing (RIP-seq) can be further investigated to accelerate the elucidation of the splicing mechanism of ZmnMAT1 on mitochondrial group II introns.

### The splicing of *Nad1* intron 1 and/or *Nad4* intron 2 requires the involvement of multiple factors

The splicing of mitochondrial group II intron is a highly complex procedure in plant, which requires the participation of diverse nuclear-encoded splicing factors, including PPR protein, transcription termination factor protein, RNA helicase, CRM domain-containing protein, PORR domain protein, RCC domain protein, RAD52-like protein, and maturase (Brown et al., 2014; Gualberto et al., 2015). In this study, we characterized a new nuclear-encoded mitochondrial type I maturase, ZmnMAT1, which is pivotal for the splicing of *Nad1* intron 1 and *Nad4* intron 2 (**Figures 5B, C**, **6**). The mutation of ZmnMAT1 impaired the assembly and activity of mitochondrial complex I (**Figure 7**), leading to delayed seed development with empty pericarp phenotype (**Figure 1**; **Supplementary Figure S1**). To date, in maize, four PPR proteins including DEK2 (Qi et al., 2017), EMP11 (Ren et al., 2017), PPR-SMR1 (Chen et al., 2019), DEK55 (Ren et al., 2020), as well as a type II maturase ZmnMAT3 (Chen et al., 2021) have been identified to involve in the splicing of *Nad1* intron 1; and PPR-SMR1 is reported to be required for the splicing of *Nad4* intron 2. Defects in each of these proteins severely disturbed the assembly and activity of mitochondrial complex I and seed development in maize.

In Arabidopsis, several splicing factors involved in the splicing of *Nad1* intron 1 and/or *Nad4* intron 2. PPR protein OTP43 is absolutely required for *trans*-splicing of *Nad1* intron 1 (de Longevialle et al., 2007). AtnMAT1, a nuclear-encoded type I maturase, functions in both the *trans*-splicing of *Nad1* intron 1 and *cis*-splicing of *Nad4* intron 2, as well as *Nad2* intron 1 (Keren et al., 2012). The mutation of AtnMAT1 led to retarded growth and a remarkable decrease in the assembly and activity of complex I (Keren et al., 2012). AtnMAT4, a nuclear-encoded type II maturase with additional D-En domain, is particularly essential for the splicing of *Nad1* intron 1, 3 and 4; defects in AtnMAT4 resulted in delayed growth and development, disturbances assembly and activity of complex I, and impaired mitochondrial morphology and function (Cohen et al., 2014). A DEXH-box RNA helicase ABO6 plays a vital role in the efficient splicing of 12 mitochondrial group II introns, including *Nad1* intron 1 and *Nad4* intron 2 (He et al., 2012). Loss of function of ABO6 displayed inhibited seed germination and primary root growth, and damaged mitochondrion function (He et al., 2012). Another DEAD-box RNA helicase protein PMH2 is essential for the splicing of 15 mitochondrial group II introns that contain *Nad4* intron 2 (Köhler et al., 2010). Surprisingly, the phenotype of the *pmh2* mutant was comparable with the WT, suggesting a possible redundant function by other mitochondrial DEAD-box proteins such as PMH1 (Köhler et al., 2010). In addition, a mitochondrial CRM domain-containing protein CFM9 is required for the efficient splicing of as many as 17 group II introns, including *Nad1* intron 1 and *Nad4* intron 2; mutation of CFM9 affects Arabidopsis growth under various abiotic stress conditions (Lee et al., 2019). In summary, the mitochondrion-localized splicing factors from diverse families in Arabidopsis display distinct differences in the specificity of intron splicing, which may be associated with their different functions in the splicing of specific group II introns.

Furthermore, Arabidopsis AtnMAT2 has been found in a large mitochondrial ribonucleoprotein complex, which also contains RNA helicase PMH2 (Zmudjak et al., 2017). In maize, PPR protein PPR-SMR1 can physically interact with CRM domain protein Zm-mCSF1 to facilitate the splicing of several mitochondrial group II introns (Chen et al., 2019). PPR14, PPR-SMR1, and Zm-mCSF1 have been reported to interact with each other to mediate the splicing of *Nad2* intron 3 (Wang et al., 2020). Recently, PPR protein EMP603 has been characterized to interact with PMH2-5140, Zm-mCSF1, ODB1-0814 and ODB1-5061, to involve in the splicing of *Nad1* intron 2 by a possible dynamic ‘spliceosome-like’ complex (Fan et al., 2021). In this study, ZmnMAT1 may not interact with DEK2, EMP11, PPR-SMR1, DEK55, and ZmnMAT3; as well as ZmnMAT4, ABO6-2858, CFM9-4040, CFM9-9857, PMH2-5140, PMH2-0512, and PMH2-7984, which are the orthologs of Arabidopsis AtnMAT4, ABO6, CFM9, and PMH2, respectively (**Figure 9**). Likewise, no direct interactions were detected by Y2H assays between two P-type PPR proteins EMP12 and EMP16 (Sun et al., 2019), as well as PPR101 and PPR231 (Yang H. et al., 2020), which are implicated in the splicing of *Nad2* intron 4, and *Nad5* intron 1 and 2, respectively. The undetectable interaction was also occurred between a PPR protein PPR20 and a mitochondrial transcription termination factors Zm_mTERF15, both of which are specifically required for the splicing of *Nad2* intron 3 in maize (Yang Y. Z. et al., 2020). Similarly, the Y2H assays and LCI assays revealed no direct interactions between type II maturase ZmnMAT3 and the 10 investigated proteins related to the splicing of *Nad1* introns (Chen et al., 2021). It is most likely that these proteins associated with each other by constituting a dynamic ‘spliceosome-like’ complex without direct interactions, or act transient interactions (that escape the detection by Y2H system) on the splicing of multiple introns. Further studies are necessary to elucidate the mechanism of the intron-splicing in organelles.

## Materials and methods

### Plant materials and growth conditions

The maize *zmnmat1* mutant was derived from a UniformMu line (UFMu-05745) requested from the Maize Genetics Cooperation Stock Center (McCarty et al., 2005). Heterozygous *zmnmat1*/*+* was crossed into B73 inbred line to generate F_1_ populations, which were used for linkage analysis. The F_1_ populations were subsequently self- pollinated to generate F_2_ ears, and the wild-type and mutant kernels from segregated F_2_ ears were used for phenotype and molecular characterization. All maize materials were grown at the experimental stations of Beijing or Hainan province, China. *Arabidopsis thaliana* (Columbia-0) and *Nicotiana benthamiana* plants were grown at 22°C under a 16 h light/8 h dark photoperiod.

### Paraffin, resin, and TEM sections

Wild-type and *zmnmat1* mutant kernels at 9 DAP and 15 DAP were collected from the same heterozygous ears, cut along the longitudinal axis, and then fixed in FAA solution (5 mL 37% formaldehyde, 90 mL 70% ethanol, and 5mL glacial acetic acid) for paraffin section preparation. The fixed samples were firstly embedded in paraffin, then cut into 8 μm sections, stained with toluidine blue, and observed under a Nikon Ti Microscope (Nikon, Tokyo, Japan) as described previously (Ren et al., 2019). For resin sections, samples at 15 DAP were fixed in 2.5% glutaraldehyde, immersed in resin, then cut into 1.4 μm sections, and observed under an Olympus IX71 Microscope. Ultrathin sections of TEM were observed under a Hitachi 7700 Transmission Electron Microscope.

### RNA extraction, RT-PCR, and qRT-PCR

For the gene expression analysis of *ZmnMAT1* in different tissues, total RNAs were extracted from various tissues of maize inbred line B73 using an RNAprep Pure Plant Kit (TianGen). For the RT-PCR analysis of the mutant and WT, total RNAs were extracted from thirty wild-type and thirty mutant kernels (without pericarps) from three independent F_2_ heterozygous ears at 10 DAP, respectively. RNA samples were digested with RNase-free DNase I (NEB) to remove residual genomic DNA. The cDNA was synthesized by using a HiScript III 1st Strand cDNA Synthesis Kit (+gDNA wiper) (Vazyme Biotech Co., Ltd) with random primers. Quantitative RT-PCR was carried out with TransStart Green qPCR SuperMix (TransGen) by a 7300-sequence detection system (Applied Biosystems). Mitochondrial gene expression and intron splicing were analyzed by RT-PCR and qRT-PCR using primer pairs reported previously (Fan et al., 2021). For each qRT-PCR sample, three technical replicates and three biological replicates were performed. *ZmActin* was served as an internal control to normalize the target gene expression. Primers are listed in **Supplementary Table S1**-**S3**.

### Phylogenetic analysis

The amino acid sequences of ZmnMATs (ZmnMAT1 to ZmnMAT4) and their homologous proteins in other five species were downloaded from MaizeGDB (https://www.maizegdb.org) and NCBI (https://blast.ncbi.nlm.nih.gov) databases, respectively. Multiple sequence alignments were performed with ClustalW1.3 program, and a phylogenetic tree was then constructed using MEGA5.1 software with neighbor-joining method.

### Targeted mutagenesis by CRISPR/Cas9 system

To confirm that *GRMZM2G023983* was *ZmnMAT1*, two 20 bp gRNA sequences (CTCTTCATTCGCTCCTCCGC and CCTTGAACCAGTCCTCGAGC) were cloned into pBUE411 to construct the pBUE411-2gRNA-*ZmnMAT1* vector (Xing et al., 2014), and then transformed into maize immature embryos of inbred line Cal as described previously (Liu et al., 2015).

### Subcellular localization

Subcellular localization was performed as described previously (Fan et al., 2021). The coding sequence of *ZmnMAT1* without the stop codon was cloned into pEarleyGate101 vector to generate a p35S::ZmnMAT1-YFP expression construct. The fusion plasmid were transiently expressed in tobacco and stably transformed into Arabidopsis, respectively. The fluorescence signals were monitored under an LSM980 confocal microscope (Zeiss, Oberkochen, Germany) with MitoTracker Red (Thermo Fisher Scientific) as a mitochondrion marker.

### Mitochondria isolation, blue-native PAGE and complex I activity assay

Mitochondria from 10 DAP kernels (without pericarps) of *zmnmat1* mutant and the WT siblings were isolated, respectively; then the blue native-polyacrylamide gel electrophoresis (BN-PAGE) and in-gel complex I activity assay were performed subsequently according to a previously reported method (Fan et al., 2021).

### Western blotting assays

Denatured mitochondrial protein extracts were separated by SDS-PAGE and then transferred to a nitrocellulose membrane (Millipore) with a semi-dry blotter (Bio-Rad). Specific antibodies including NAD9 (PhytoAB, PHY0516S), Cyt*c* (Agrisera, AS08343A), COX2 (Agrisera, AS04053A), ATPase-B (Agrisera, AS05085), and AOX (PhytoAB, PHY1404S), and the SuperSignal™ West Pico PLUS Chemiluminescent Substrate Kit (Thermo Fisher Scientific) were used to examine the protein level changes between WT and *zmnmat1* mutant as described previously (Chen et al., 2021; Fan et al., 2021).

### Yeast two-hybrid assays

The Y2H assays were performed according to the Matchmaker™ Gold Yeast Two-Hybrid System (Clontech) manul. For bait, the full-length coding sequence (with stop codon) of ZmnMAT1 was recombined into the GAL4 DNA-binding domain vector at *Eco*RI and *Bam*HI restriction sites to generate pGBKT7-ZmnMAT1. For preys, the full-length open reading frames of DEK2, EMP11, Zm-mCSF1, PPR-SMR1, ABO6-2858, CFM9-4040, CFM9-9857, ZmnMAT4, PMH2-5140, PMH2-7984, PMH2-0512, and DEK55 were fused downstream of the GAL4 activation domain at *Eco*RI and *Bam*HI sites to produce pGADT7-preys. Different combinations of pGBKT7-ZmnMAT1 and pGADT7-preys plasmids were co-transformed into yeast strain Y2H Gold (Clontech) by a Frozen-EZ Yeast Transformation II Kit (MKbio). Co-transformed cells were incubated on SD/-Leu/-Trp (DDO) dropout medium and SD/-Ade/-His/-Leu/-Trp dropout supplemented with AbA (QDO+AbA) medium with a dilution series for 4 days at 30°C to verify protein-protein interactions. The interaction between BD-P53 and AD-T was used as a positive control. The interactions between BD-lam and AD-T, as well as pGBKT7-ZmnMAT1 and pGBKT7-empty were used as negative controls. All primers used are listed in **Supplementary Table S3**.

### Accession numbers

Sequence information from this article can be found in the NCBI data libraries under the following accession numbers: ZmnMAT1, GRMZM2G023983/XP_008651784.1; ZmnMAT2, GRMZM2G154119/XP_020400703.1; ZmnMAT3, AC213050.3_FG001/XP_020394952.1; ZmnMAT4, GRMZM2G375999/XP_008659802.1; AtnMAT1, NP_174294.1; AtnMAT2, NP_199503.1; AtnMAT3, NP_001154695.1; AtnMAT4, NP_177575.1; OsnMAT1, XP_025877888.1; OsnMAT2, XP_025876488.1; OsnMAT3, XP_015640954.1; OsnMAT4, XP_015643434.1; GmnMAT1, XP_003547207.2; GmnMAT2, XP_003534769.1; GmnMAT3, XP_025981807.2; GmnMAT4, XP_006600812.1; BdnMAT1, XP_014752427.1; BdnMAT2, XP_014757199.1; BdnMAT3, XP_003560393.2; BdnMAT4, XP_024312252.1; GhnMAT1, XP_016722554.2; GhnMAT2, XP_040960639.1; GhnMAT3, XP_016669292.1; GhnMAT4, XP_016715130.1; AOX1, AY059646.1; AOX2, AY059647.1; AOX3, AY059648.1.

## Data availability statement

The datasets presented in this study can be found in online repositories. The names of the repository/repositories and accession number(s) can be found in the article/**Supplementary Material**.

## Author contributions

YJL, KF, and ZR designed the experiments. KF, ZR, QF, QW, SJ, AZ, TW, JC, and YL, performed the experiments. KF, ZR, and YJL analyzed the data. KF, ZR, and YJL wrote the article. All authors contributed to the article and approved the submitted version.

## Funding

This work was supported by the National Natural Science Foundation of China (31871631) and the Agricultural Science and Technology Innovation Program of CAAS.

## Acknowledgments

We thank the Maize Genetics Cooperation Stock Center for providing the maize stock UFMu-05745.

## Conflict of interest

The authors declare that the research was conducted in the absence of any commercial or financial relationships that could be construed as a potential conflict of interest.

## Publisher’s note

All claims expressed in this article are solely those of the authors and do not necessarily represent those of their affiliated organizations, or those of the publisher, the editors and the reviewers. Any product that may be evaluated in this article, or claim that may be made by its manufacturer, is not guaranteed or endorsed by the publisher.
