# Supplementary material for: ZmnMAT1, a nuclear-encoded type I maturase, is required for the splicing of mitochondrial Nad1 intron 1 and Nad4 intron 2
Source: Front Plant Sci. 2022 Nov 23;13:1033869. doi: 10.3389/fpls.2022.1033869 (PMC9727264; doi:10.3389/fpls.2022.1033869)
Supplement: Supplementary file 1 [file DataSheet_1.docx]

| **Table S1**. Primers used for RT-PCR analysis of 35 mitochondrial genes.   \| Primer name \| Primer sequence 5'-->3' \| \| --- \| --- \| \| nad1-F1 \| GGCCCGATCATGAGTGAATA \| \| nad1-R1 \| GCCCCCTTCAGAAGAAACTT \| \| nad2-F1 \| GACGGAGGAGAGGAAATGAA \| \| nad2-R1 \| GCAGTCCACCCTTTCTTTGA \| \| nad3-F1 \| CTTTCCTATGTCCTTCCCCC \| \| nad3-R1 \| GAGGAGAGCGAGAGAACGAA \| \| nad4-F1 \| CAGTCACCCGGAGAAGATTT \| \| nad4-R1 \| TAATTTGGCGCCTGATTGAC \| \| nad4L-F1 \| CTGACATTCCATGTTTCCGA \| \| nad4L-R1 \| GAAGAGAACGAAAGGAGAACAGA \| \| nad5-F1 \| CGCTCGAACATTGTCTGATT \| \| nad5-R1 \| GTCCTGGCAAGCTCCTACAG \| \| nad6-F1 \| TGGAAAAACCAAACCCACAT \| \| nad6-R1 \| CAAGTTCCCTTGGCGTAGTC \| \| nad7-F1 \| GTTTTGGCTCGCAATAAAGC \| \| nad7-R1 \| CAGGTGGGACAAGCTCTAGG \| \| nad9-F1 \| AGCAAGAAGCGGAACAAAAA \| \| nad9-R1 \| TATTGATTTGTCCCCTCCCC \| \| rps1-F1 \| AAGGTGGGCTTCGGATTATT \| \| rps1-R1 \| TCTTCAGTTTTACGCTTACGCT \| \| rps2A-F1 \| CAGGAAAGATATTTGCCCCA \| \| rps2A-R1 \| CCTGTATCTCCGGAAACGAA \| \| rps2B-F1 \| TCCATGGACCCACGTAAAAT \| \| rps2B-R1 \| GGCCCCTCTCTGATAAGGAA \| \| rps3-F1 \| GCAGAAAGGGGCAAAAGTAA \| \| rps3-R1 \| TCGCGACCCCTACTACATCT \| \| rps4-F1 \| AGAGTTGGGTTCGATTCCCT \| \| rps4-R1 \| AGCGACTAGGCCGATCTTTT \| \| rps7-F1 \| TTCGTTGGAAAAACCTACGC \| \| rps7-R1 \| ATGAGGAAGGCCGATTTTCT \| \| rps7-ct-F1 \| TTGAACCTCTTTCACGCTCA \| \| rps7-ct-R1 \| TTCCGATCGAGATGTATGGA \| \| rps12-F1 \| CTAGCTGCTTCCATATCGCC \| \| rps12-R1 \| CGGATCGGGAGTAACCACTA \| \| rps12-ct-F1 \| TGTACGGTTCTGTAGAGGGACA \| \| rps12-ct-R1 \| TCCGTTTTCTTTTTATAAGGGC \| \| rps13-F1 \| TCATGATGATTAAGGGAAGAGTGA \| \| rps13-R1 \| TTGAATTGAACAGTGTGATTGAT \| \| rpl16-F1 \| GGTTTTTCCCCACTAACCAA \| \| rpl16-R1 \| GGGTGCGGAAATAGCTAGAA \| \| atp1-F1 \| CGTTGCTGGTGAAGAAGCAT \| \| atp1-R1 \| AAAAGCGGATTTATCCATCG \| \| atp4-F1 \| AGCCACGTGCTCTAATCCTC \| \| atp4-R1 \| TCCCTTTCTCTTGGAGCAGA \| \| atp6-F1 \| CCAAGTCTCTTTTGGGAGCA \| \| atp6-R1 \| GGCTCCTCGTTTTTATGCAA \| \| atp8-F1 \| GGCAAGGATCCTCAGTCCTA \| \| atp8-R1 \| GAGGGTTGGTTTGATTGGAA \| \| atp9-F1 \| AGGGGCCTCGTCATCTCTAT \| \| atp9-R1 \| TAGTTGCGAAGGAAAAGCGT \| \| ccmB-F1 \| AGCCGTCGAAGTGAATGAAT \| \| ccmB-R1 \| AACGGCTTTTCCATGACTTG \| \| ccmC-F1 \| ACTTGCAAGGCAAGGAAAAA \| \| ccmC-R1 \| CCATGGATGCTTTAGCGAGT \| \| ccmF_C_-F1 \| GAGAAGCTCAAATCGAACGG \| \| ccmF_C_-R1 \| CGCAGCCACTATTTTGACTC \| \| ccmF_N_-F1 \| TGAAGATTGTAAGGCGTTTCC \| \| ccmF_N_-R1 \| GGATCATCCTGTGGTTACCG \| \| cob-F1 \| ATCAAGGCAAGGGGGTAAAT \| \| cob-R1 \| GGTGTGATCAGTCTCATCCG \| \| cox1-F1 \| GGCCCCTCTCTGATAAGGTT \| \| cox1-R1 \| GTTAAGGCAAAGCCCAAACA \| \| cox2-F1 \| GTCCTACTTCTGGTGCTGCC \| \| cox2-R1 \| GAGAATTGCATTTCCGCTTC \| \| cox3-F1 \| TCAATCCACTTATTCGTTCCC \| \| cox3-R1 \| GTTTACATACAACCGGGGCA \| \| mat-R-F1 \| AACGCCTGTTCGCTAAAATC \| \| mat-R-R1 \| AGGCTTTGCTCCCCTTTTT \| \| mttB-F1 \| TTGGTTTAGAATTGCTCGGG \| \| mttB-R1 \| AGGGGGAACCCTACCGAC \|   **Table S2.** Primers used for qRT-PCR and RT-PCR analysis of mitochondrial intron splicing.   \| Primer name \| Primer sequence 5'-->3' \| \| --- \| --- \| \| nad1 exon1F \| GCAACGTCGAAAGGGTCCTG \| \| nad1 exon2R \| TGAGCTGCAGATCGTAATGC \| \| nad1 intron1 exon1F \| CAAGTTGGGTTGGGGTATAGAG \| \| nad1 intron1 exon1R \| TGAGCTGCAGATCGTAATGC \| \| nad1 exon2F \| TCGAAATATGCCTTTCTAGGAG \| \| nad1 exon3R \| ATTCAGCTTCCGCTTCTGG \| \| nad1 intron2 exon2F \| CTCGGATAAAAGCACGGACGAG \| \| nad1 intron2 exon2R \| ATTCAGCTTCCGCTTCTGG \| \| nad1 exon3F \| GTCATGGCGCAAAAGCAGATATGG \| \| nad1 exon4R \| AGAGCAGACCCCATTGAAGA \| \| nad1 intron3 exon3F \| GTCGATTTATCCACACTTCCATGAC \| \| nad1 intron3 exon3R \| AGAGCAGACCCCATTGAAGA \| \| nad1 exon4F \| TCTTCAATGGGGTCTGCTCT \| \| nad1 exon5R \| AGGGAGCCATCGAAAGGTGA \| \| nad1 intron4 exon4F \| TCTTCAATGGGGTCTGCTCT \| \| nad1 intron4 exon4R \| GACCACCTATCACTAGTCTTCGG \| \| nad2 exon1F \| GACGGAGGAGAGGAAATGAA \| \| nad2 exon2R \| GCCGGGATCATTAAGAGCATAC \| \| nad2 intron1 exon1F \| GACGGAGGAGAGGAAATGAA \| \| nad2 intron1 exon1R \| CAACCTTACTCATGGCAACCTTC \| \| nad2 exon2F \| CTCGCAGTATGCTCTTAATGATCC \| \| nad2 exon3R \| GGAACTGCAGTAATCTTGAATAGGG \| \| nad2 intron2 exon2F \| CTCGCAGTATGCTCTTAATGATCC \| \| nad2 intron2 exon2R \| CAACCTTACTCATGGCAACCTTC \| \| nad2 exon3F \| TCTACTGGAGCTACCCACTTCGA \| \| nad2 exon4R \| GGTTTGCCGTAATGCTGGA \| \| nad2 intron3 exon3F \| TCTACTGGAGCTACCCACTTCGA \| \| nad2 intron3 exon3R \| CAACCTTACTCATGGCAACCTTC \| \| nad2 exon4F \| TTCCAGCATTACGGCAAACC \| \| nad2 exon5R \| GCAGTCCACCCTTTCTTTGA \| \| nad2 intron4 exon4F \| TTCCAGCATTACGGCAAACC \| \| nad2 intron4 exon4R \| CAACCTTACTCATGGCAACCTTC \| \| nad4 exon1F \| GGTCCTATTCTCTGTCCCGTGC \| \| nad4 exon2R \| GTAAATCGGTGGTTCCTGTTTGG \| \| nad4 intron1 exon1F \| CTGGAGCTTCGACCAACCAC \| \| nad4 intron1 exon1R \| GTAAATCGGTGGTTCCTGTTTGG \| \| nad4 exon2F \| TCATTATAGGGGTATGGGGTTCG \| \| nad4 exon3R \| CTAGTGCCGGGTAAACTCATATTG \| \| nad4 intron2 exon2F \| GTAACTATCTTGTACGGTTCGGG \| \| nad4 intron2 exon2R \| CTAGTGCCGGGTAAACTCATATTG \| \| nad4 exon3F \| TAGTCCGAACATACCGGGAATTG \| \| nad4 exon4R \| CTTACGGATGTATGCATGCAGTC \| \| nad4 intron3 exon3F \| GAGACTATCTAGCTTGGTTCGGAG \| \| nad4 intron3 exon3R \| CTTACGGATGTATGCATGCAGTC \| \| nad5 exon1F \| CGCTCGAACATTGTCTGATT \| \| nad5 exon2R \| AGCAGATACTGGAGTGGGAC \| \| nad5 intron1 exon1F \| CATCGGAAATGTTTGATGCTTCTTG \| \| nad5 intron1 exon1R \| GATTCCCGACATGCTATGATACCC \| \| nad5 exon2F \| GTCACTGCTGGCGTTTTC \| \| nad5 exon3R \| TACCTAAACCAATCATCATATC \| \| nad5 intron2 exon2F \| TAATTCATTCGGGCGAGACAGATTA \| \| nad5 intron2 exon2R \| ATGATCGTGTTGGGTAAATTGTGAC \| \| nad5 exon3F \| GATATGATGATTGGTTTAGGTA \| \| nad5 exon4R \| GCCAATCGTCGGAATGTG \| \| nad5 intron3 exon3F \| GATATGATGATTGGTTTAGGTA \| \| nad5 intron3 exon3R \| TATGTTTCCCTTCTTCCATTCTCCA \| \| nad5 exon4F \| TTGCCGAATCCGAGTTTG \| \| nad5 exon5R \| GTCCTGGCAAGCTCCTACAG \| \| nad5 intron4 exon4F \| GTGGTAAAGGGAGGGAGGATATTAG \| \| nad5 intron4 exon4R \| GTCCTGGCAAGCTCCTACAG \| \| nad7 exon1F \| TAATTTGGCGCCTGATTGAC \| \| nad7 exon2R \| CTCGATTAATTTCTCAGTCCCTC \| \| nad7 intron1 exon1F \| AGCTCTAGGGGAATAATCTCTTTCT \| \| nad7 intron1 exon1R \| CAAAGTCAGGTAGAGCGGTTCG \| \| nad7 exon2F \| GCCTCTTGGCTTATGTCGAG \| \| nad7 exon3R \| CCGAACACTTTGTCGCATCT \| \| nad7 intron2 exon2F \| GAGGGACTGAGAAATTAATCGAG \| \| nad7 intron2 exon2R \| CTGTAGTCGTCGCCATATTCAATAT \| \| nad7 exon3F \| GAGGGACTGAGAAATTAATCGAG \| \| nad7 exon4R \| CTCGACATAAGCCAAGAGGC \| \| nad7 intron3 exon3F \| GCCTCTTGGCTTATGTCGAG \| \| nad7 intron3 exon3R \| ATATGCATGCTTTTGTAGGGTCG \| \| nad7 exon4F \| AGATGCGACAAAGTGTTCGG \| \| nad7 exon5R \| GTTTTGGCTCGCAATAAAGC \| \| nad7 intron4 exon4F \| AGATGCGACAAAGTGTTCGG \| \| nad7 intron4 exon4R \| ATGCTTTACTCCTAACCCCACG \| \| cox2 exon1F \| GTCCTACTTCTGGTGCTGCC \| \| cox2 exon2R \| GAGAATTGCATTTCCGCTTC \| \| cox2 intron1 exon1F \| GCTCTGTTATACTCAATGGACGGG \| \| cox2 intron1 exon1R \| GAGCATTTCGGGGTATAGGTCTAA \| \| rps3 exon1F \| GCAGAAAGGGGCAAAAGTAA \| \| rps3 exon1R \| CAGAGCGGGACTTCTTTGGTA \| \| rps3 intron1 exon1F \| TTTCGGTAAGACTTGATCTGAATCG \| \| rps3 intron1 exon1R \| TCTAATTCTAAACCCATTGTCGTCC \| \| ccmF_C_ exon1F \| CGATAGGTCAGCGAAGCGTG \| \| ccmF_C_ exon1R \| AGACCTCGCAAACAACAACGT \| \| ccmF_C_ intron1 exon1F \| CTCCCCATCTCTCCTCAACTTC \| \| ccmF_C_ intron1 exon1R \| AGACCTCGCAAACAACAACGT \|  \| **Table S3**. Primers used in *ZmnMAT1* analysis. \| \| \| \| --- \| --- \| --- \| \| Primer name \| Primer sequence 5' to 3' \| Use for \| \| TIR5 \| GCTCTTCKTCYATAATGRC  AATT \| Identify *Mu* insertion site and genotype in *zmnmat1* mutant \| \| TIR6 \| AGAGAAGCCAACGCCAWC  GCCTCYATTTCGTC \| \| TIR8.1 \| CGCCTCCATTTCGTCGAAT  CCCCTS \| \| TIR8.2 \| CGCCTCCATTTCGTCGAAT  CCSCTT \| \| TIR8.3 \| SGCCTCCATTTCGTCGAAT  CCCCKT \| \| TIR8.4 \| CGCCTCCATTTCGTCGAAT  CACCTC \| \| nMAT1-05745F0.7 \| ACTACCCACCACCAATTCGT \| \| nMAT1-05745R0.7 \| TCTCGCTGACAATGTTCCCT \| \| nMAT1-qF3 \| TCACCGATGCTGTACTCTGG \| qRT-PCR analysis of *ZmnMAT1* gene expression \| \| nMAT1-qR3 \| GCTTTCTCTCAAAGGACGGC \| \| pGWC-nMAT1-F \| AGCAGGCTTTGACTTTATGCC  GCCGCGCGCG \| Construct ZmnMAT1:YFP vector \| \| pGWC-nMAT1 (-TGA) \| TGGGTCTAGAGACTTTccTGTA  TCCAATAGAAGTTGCTTGTTC \| \| ZmAox1-qF \| CCTATTGGACCGTCAAATTACTGC \| qRT-PCR analysis of *Aox* gene expression \| \| ZmAox1-qR \| CACTGTTTCCAGCATCATAGCAC \| \| ZmAox2-qF \| CCAAGACGCTGATGGATAAGGT \| \| ZmAox2-qR \| CCACGGTTTCCAGCATCAT \| \| ZmAox3-qF \| CGGCACCGAGAAGCATGA \| \| ZmAox3-qR \| CTGGTCCACTTCCACTCCGT \| \| ZmActin-qF \| ATGGTCAAGGCCGGTTTCG \| \| ZmActin-qR \| TCAGGATGCCTCTCTTGGCC \| \| AD-nMAT4-F \| GGAGGCCAGTGAATTCATGA  CGCGTGTGGCAGC \| Construct AD vectors \| \| AD-nMAT4-R \| CGAGCTCGATGGATCCTCATCT  AATCAGGGAACCAAAAT \| \| AD-PMH2-7984-F \| GGAGGCCAGTGAATTCATGTA  CGCCGTCCTCCGC \| \| AD-PMH2-7984-R \| CGAGCTCGATGGATCCCTACC  GTGAGTTCTTGTCGCC \| \| AD-PMH2-5140-F \| GGAGGCCAGTGAATTCATGCA  AGGAAGGGACATGGTT \| \| AD-PMH2-5140-R \| CGAGCTCGATGGATCCCTATCG  TGAGTCCTTGTCGCC \| \| AD-ABO6-2858F \| GGAGGCCAGTGAATTCATGAG  CGCTACTCTAAACGCG \| \| AD-ABO6-2858R \| CGAGCTCGATGGATCCTCATTT  CCATCTAATGCTCCAATC \| \| AD-DEK2-F \| GGAGGCCAGTGAATTCATGCCC  CATCCCGCCA \| \| AD-DEK2-R \| CGAGCTCGATGGATCCCTAAGGC  CTCCAAATCTCATCCC \| \| AD-DEK55-F \| GGAGGCCAGTGAATTCGACCGCT  ACCTTGCTAACGCG \| \| AD-DEK55-R \| CGAGCTCGATGGATCCTCAGATAC  CAAAATCACAAGC \| \| AD-CFM9-4040-F \| GGAGGCCAGTGAATTCATGTG  GAGCCTCCTACGGG \| \| AD-CFM9-4040-R \| CGAGCTCGATGGATCCTCAGT  CACTATCCCAACTTTCATCA \| \| AD-CFM9-9857-F \| GGAGGCCAGTGAATTCATGTG  GGTTCTCCGGAGC \| \| AD-CFM9-9857-R \| CGAGCTCGATGGATCCTCAGT  CACTGTCCCAACTTTCAT \| \| AD-EMP11-F \| GGAGGCCAGTGAATTCATGTC  CCTCGCGGGGG \| \| AD-EMP11-R \| CGAGCTCGATGGATCCTCAGG  AACCAGCGAATCTCTG \| \| AD-PPR-SMR1-F \| GGAGGCCAGTGAATTCATGCT  GCTCCGCGTTGG \| \| AD-PPR-SMR1-R \| CGAGCTCGATGGATCCTCACC  TAGGCATGCCAAGG \| \| AD-PMH2-8787-F \| GGAGGCCAGTGAATTCATGAT  CTCCCTGCTCCGAC \| \| AD-PMH2-8787-R \| CGAGCTCGATGGATCCTCAAC  GCAAATAATAAAAGAAGTCA \| \| AD-nMAT3-F \| GGAGGCCAGTGAATTCATGCTC  CACCGCCTCGCCCACA \| \| AD-nMAT3-R \| CGAGCTCGATGGATCCTCATTCC  ACATCAACAAATTGAGTG \| \| BD-nMAT1-F \| CATGGAGGCCGAATTCATGC  CGCCGCGCGCG \| Construct BD vector \| \| BD-nMAT1-R \| GCAGGTCGACGGATCCCTATG  TATCCAATAGAAGTTGCTTGTTC \| \| ZmAox1-RT-F \| AAGGTGCTGCTCGACAAGAT \| RT-PCR analysis of *Aox* gene expression \| \| ZmAox1-RT-R \| GTAGGCGTTGAAGAAGACGC \| \| ZmAox2-RT-F \| GACATCTTCTTCCAGAGGCG \| \| ZmAox2-RT-R \| TGACTACGTCCTTGAGCGTG \| \| ZmAox3-RT-F \| CCAAGCTCGTGAAGGAAGAC \| \| ZmAox3-RT-R \| GTAGGCGTTGAAGAAGACGC \| |
| --- | --- | --- | --- | --- | --- | --- | --- | --- | --- | --- | --- | --- | --- | --- | --- | --- | --- | --- | --- | --- | --- | --- | --- | --- | --- | --- | --- | --- | --- | --- | --- | --- | --- | --- | --- | --- | --- | --- | --- | --- | --- | --- | --- | --- | --- | --- | --- | --- | --- | --- | --- | --- | --- | --- | --- | --- | --- | --- | --- | --- | --- | --- | --- | --- | --- | --- | --- | --- | --- | --- | --- | --- | --- | --- | --- | --- | --- | --- | --- | --- | --- | --- | --- | --- | --- | --- | --- | --- | --- | --- | --- | --- | --- | --- | --- | --- | --- | --- | --- | --- | --- | --- | --- | --- | --- | --- | --- | --- | --- | --- | --- | --- | --- | --- | --- | --- | --- | --- | --- | --- | --- | --- | --- | --- | --- | --- | --- | --- | --- | --- | --- | --- | --- | --- | --- | --- | --- | --- | --- | --- | --- | --- | --- | --- | --- | --- | --- | --- | --- | --- | --- | --- | --- | --- | --- | --- | --- | --- | --- | --- | --- | --- | --- | --- | --- | --- | --- | --- | --- | --- | --- | --- | --- | --- | --- | --- | --- | --- | --- | --- | --- | --- | --- | --- | --- | --- | --- | --- | --- | --- | --- | --- | --- | --- | --- | --- | --- | --- | --- | --- | --- | --- | --- | --- | --- | --- | --- | --- | --- | --- | --- | --- | --- | --- | --- | --- | --- | --- | --- | --- | --- | --- | --- | --- | --- | --- | --- | --- | --- | --- | --- | --- | --- | --- | --- | --- | --- | --- | --- | --- | --- | --- | --- | --- | --- | --- | --- | --- | --- | --- | --- | --- | --- | --- | --- | --- | --- | --- | --- | --- | --- | --- | --- | --- | --- | --- | --- | --- | --- | --- | --- | --- | --- | --- | --- | --- | --- | --- | --- | --- | --- | --- | --- | --- | --- | --- | --- | --- | --- | --- | --- | --- | --- | --- | --- | --- | --- | --- | --- | --- | --- | --- | --- | --- | --- | --- | --- | --- | --- | --- | --- | --- | --- | --- | --- | --- | --- | --- | --- | --- | --- | --- | --- | --- | --- | --- | --- | --- | --- | --- | --- | --- | --- | --- | --- | --- | --- | --- | --- | --- | --- | --- | --- | --- | --- | --- | --- | --- | --- | --- | --- | --- | --- | --- | --- | --- | --- | --- | --- | --- | --- | --- | --- | --- | --- | --- | --- | --- | --- | --- | --- | --- | --- | --- | --- | --- | --- | --- | --- | --- | --- | --- | --- | --- | --- | --- | --- | --- | --- | --- | --- | --- | --- | --- | --- | --- | --- | --- | --- | --- | --- | --- | --- | --- | --- | --- | --- | --- | --- | --- | --- | --- | --- | --- | --- | --- | --- | --- | --- | --- | --- | --- | --- | --- | --- | --- | --- | --- | --- | --- | --- | --- | --- | --- | --- | --- | --- |


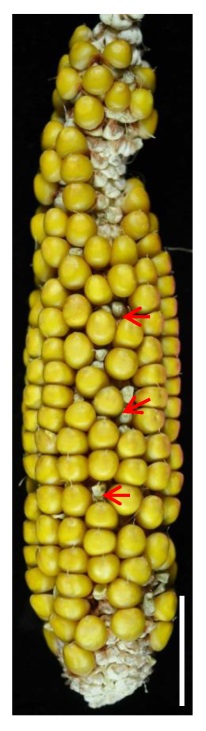


**Fig. S1. Phenotype of self-pollinated *zmnmat1*/+ segregating ear.**

The red arrows identify the *zmnmat1*mutant seeds. Scale bar = 2 cm.


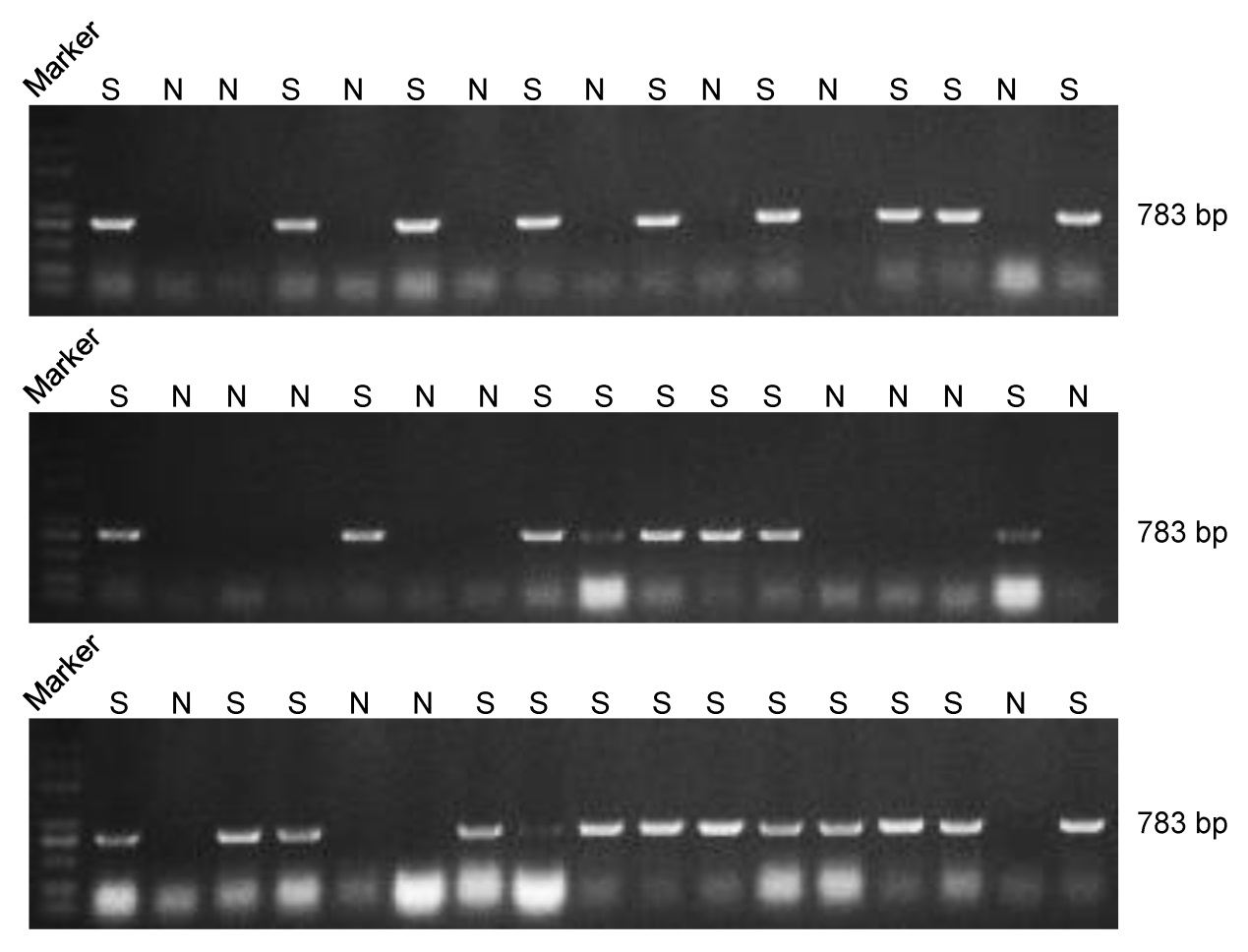


**Fig.S2. Mutations in *ZmnMAT1* are linked to the empty pericarp phenotype in *zmnmat1* mutant.**

Genotype analysis of *zmnmat1* mutant alelle. Individual plants of F_1_ population were genotyped with *ZmnMAT1* specific primer and *Mu*-specific primers. PCR bands at 783 bp is indicative of *zmnmat1*. The *emp* phenotype of each individual plant was determined by analyzing the ear for segregation after selfing. N, non-segregating (the wild type, genotype is +/+); S, segregating (heterozygous, genotype is *zmnmat1* /+).


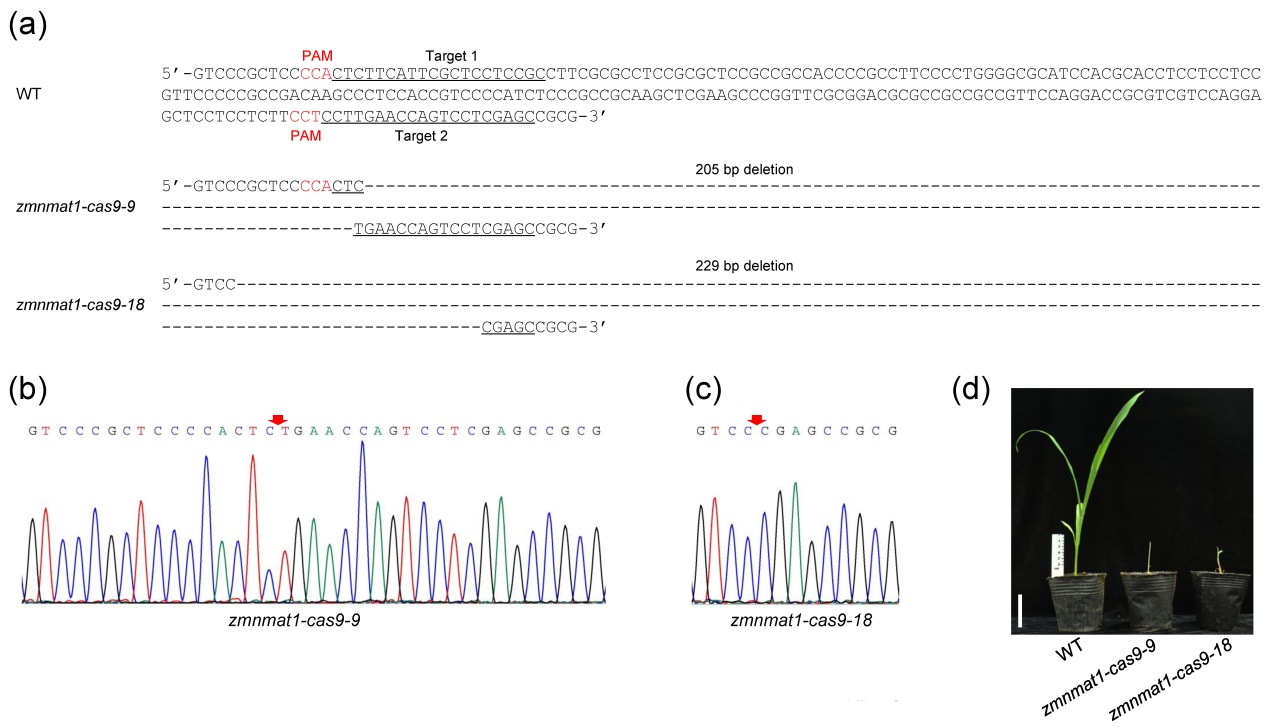


**Fig.S3. The sequence in the GRMZM2G023983 locus is targeted using CRISPR/Cas9.**

**(a)** The two gRNA sequences (Target 1 and Target 2) are underlined, the protospacer adjacent motif (PAM) are shown in red letter and the dashes represent deletions. Alignments of mutant sequences from two independent T_0_ transgenic lines are indicated.

**(b)** The genotype analysis of *zmnmat1-cas9-9* mutant. The red arrow indicates the deletion site in *zmnmat1-cas9-9* mutant.

**(c)** The genotype analysis of *zmnmat1-cas9-18* mutant. The red arrow indicates the deletion site in *zmnmat1-cas9-18* mutant.

**(d)** Phenotypes of WT and the two edited plants (*zmnmat1-cas9-9* and *zmnmat1-cas9-18*). Scale bar = 6 cm.


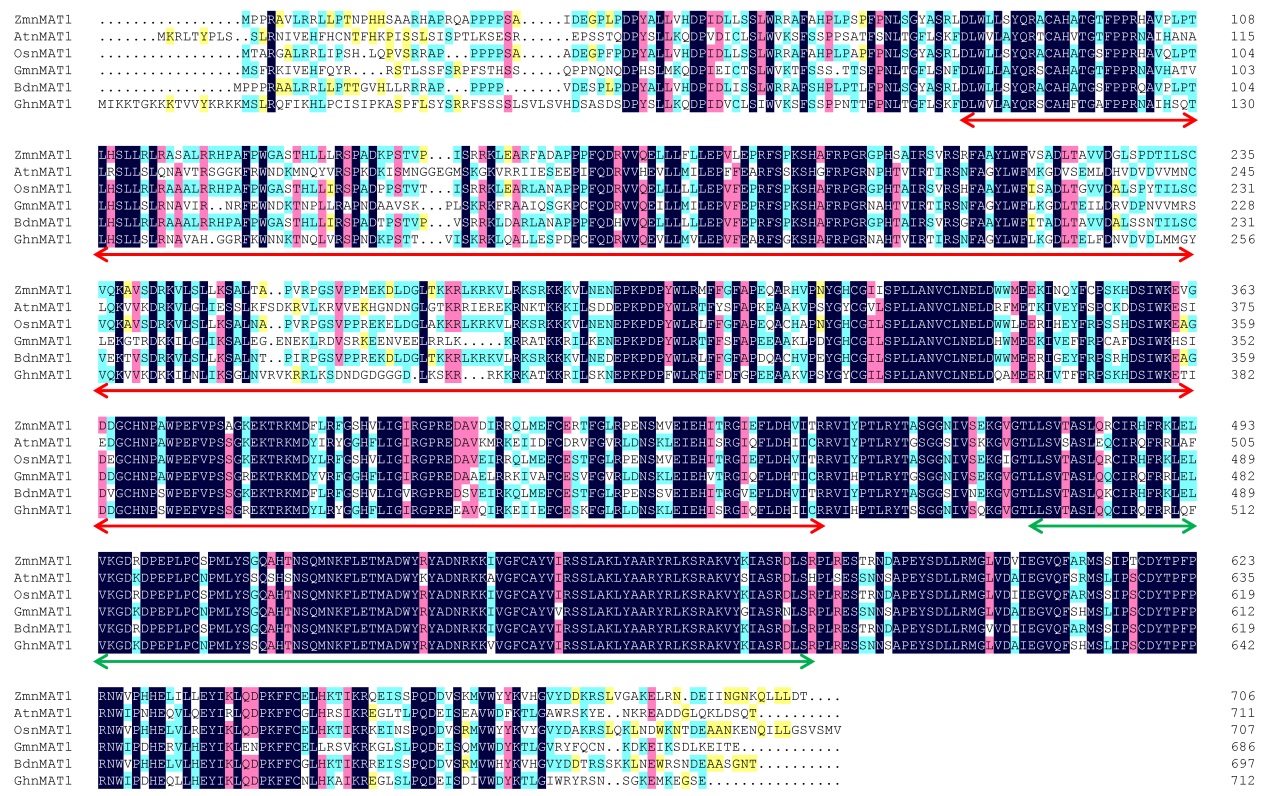


**Fig.S4. Amino acid alignment** **of ZmnMAT1 homologs.**

The amino acid sequence of ZmnMAT1 homologs, including the *Arabidopsis thaliana* (AtnMAT1), *Oryza sativa* (OsnMAT1), *Glycine max* (GmnMAT1), *Brachypodium distachyon* (BdnMAT1), and *Gossypium hirsutum* (GhnMAT1) was downloaded from Phytozome v.12.1 ([https://phytozome.jgi.doe.gov](https://phytozome.jgi.doe.gov/)) and was aligned with DNAMAN. Red double arrows indicate the RT motif and green double arrows indicate the X motif.


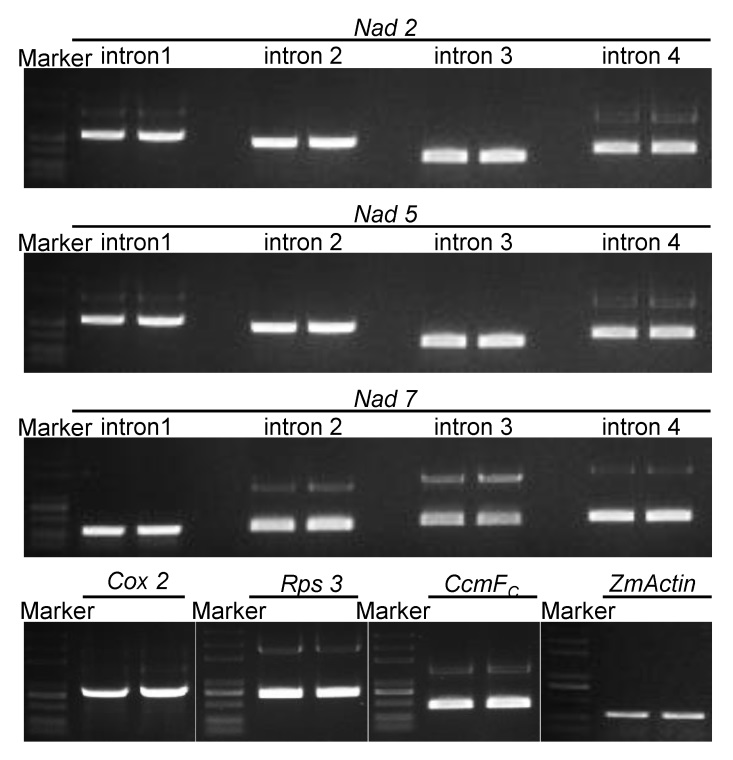


**Fig. S5. *ZmnMAT1* is specifically involved in the splicing of *Nad1* intron 1 and *Nad4* intron 2.**

RT-PCR analysis of the remaining 15 group II introns in maize mitochondrial genes was performed with RNA isolated from *zmnmat1* and WT kernels. In each gel of the introns, the two lanes are WT and *zmnmat1* mutant, respectively. *ZmActin* was used as an internal control.


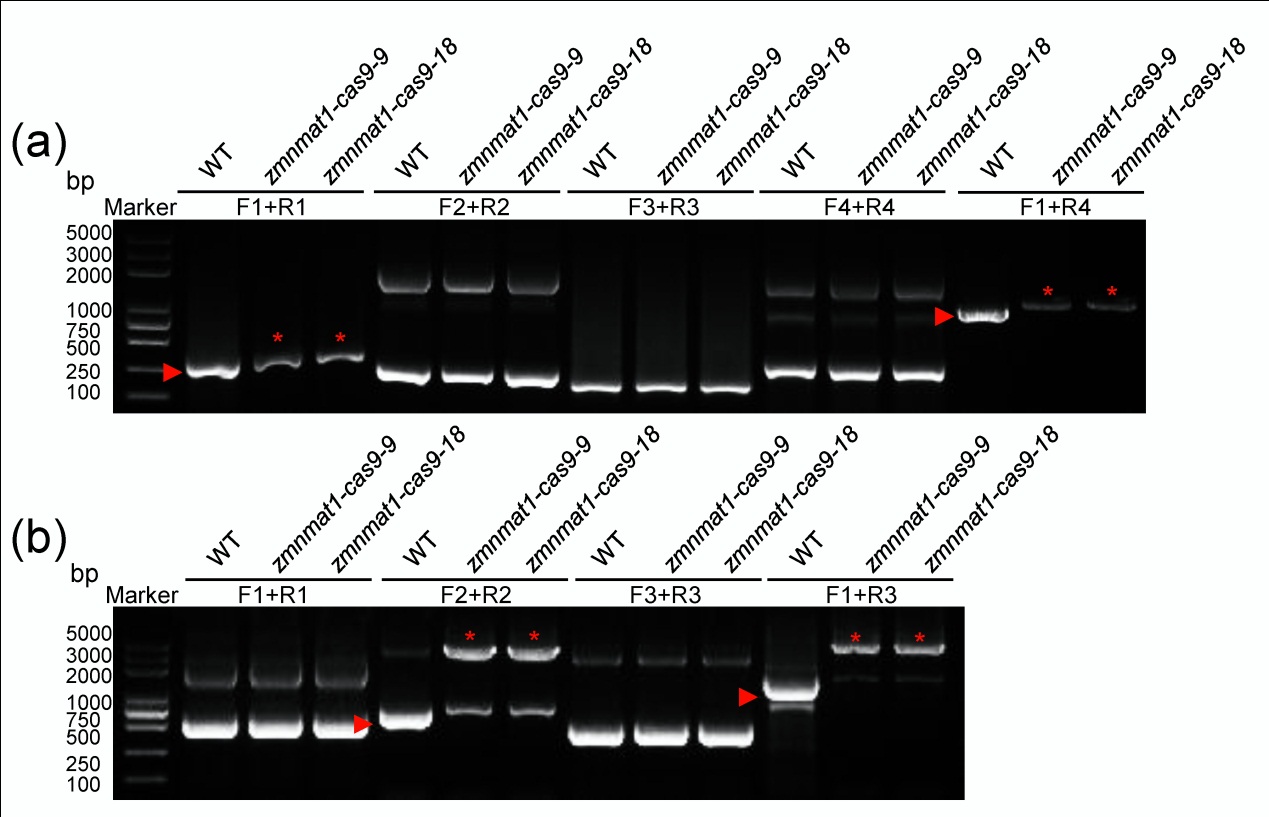


**Fig. S6. The splicing deficiency of *Nad1* intron 1 and *Nad4* intron 2 in *zmnmat1-cas9-9* and *zmnmat1-cas9-18* mutants***.*

**(a)** RT-PCR analysis of *Nad1* intron-splicing efficiency in WT and two independent T_0_ gene-edited lines *zmnmat1-cas9-9* and *zmnmat1-cas9-18*. The expected amplification products using different primer pairs are indicated as Fig.5b. (Red asterisks indicate the abnormal unspliced fragments and red triangles indicate the normal spliced fragments).

**(b)** RT-PCR analysis of *Nad4* intron-splicing efficiency in WT and two independent T_0_ gene-edited lines *zmnmat1-cas9-9* and *zmnmat1-cas9-18*. The expected amplification products using different primer pairs are indicated as Fig.5c. (Red asterisks indicate the abnormal unspliced fragments and red triangles indicate the normal spliced fragments).
